# Advancing drug–target interaction prediction: a comprehensive graph-based approach integrating knowledge graph embedding and ProtBert pretraining

**DOI:** 10.1186/s12859-023-05593-6

**Published:** 2023-12-19

**Authors:** Warith Eddine Djeddi, Khalil Hermi, Sadok Ben Yahia, Gayo Diallo

**Affiliations:** 1 LR11ES14, Faculty of Sciences of Tunis, University of Tunis El Manar, Campus Universitaire, 2092 Tunis, Tunisia; 2 High Institute of Informatics in Kef, University of Jendouba, Saleh Ayech, 8189 Jendouba, Tunisia; 3 Department of Software Science, Tallinn University of Technology, Ehitajate tee-5, 12618 Tallinn, Estonia; 4 Bordeaux Population Health Inserm 1219, University of Bordeaux, rue Léo Saignat, 33000 Bordeaux, France; 5 The Maersk Mc-Kinney Moller Institute, Southern Syddansk Universitet, Alsion 2, 6400 Sønderborg, Denmark

**Keywords:** Drug–target interaction prediction, Knowledge graph embedding, COVID-19, Cosine similarity, ProtBERT

## Abstract

**Background:**

The pharmaceutical field faces a significant challenge in validating drug target interactions (DTIs) due to the time and cost involved, leading to only a fraction being experimentally verified. To expedite drug discovery, accurate computational methods are essential for predicting potential interactions. Recently, machine learning techniques, particularly graph-based methods, have gained prominence. These methods utilize networks of drugs and targets, employing knowledge graph embedding (KGE) to represent structured information from knowledge graphs in a continuous vector space. This phenomenon highlights the growing inclination to utilize graph topologies as a means to improve the precision of predicting DTIs, hence addressing the pressing requirement for effective computational methodologies in the field of drug discovery.

**Results:**

The present study presents a novel approach called DTIOG for the prediction of DTIs. The methodology employed in this study involves the utilization of a KGE strategy, together with the incorporation of contextual information obtained from protein sequences. More specifically, the study makes use of Protein Bidirectional Encoder Representations from Transformers (ProtBERT) for this purpose. DTIOG utilizes a two-step process to compute embedding vectors using KGE techniques. Additionally, it employs ProtBERT to determine target–target similarity. Different similarity measures, such as Cosine similarity or Euclidean distance, are utilized in the prediction procedure. In addition to the contextual embedding, the proposed unique approach incorporates local representations obtained from the Simplified Molecular Input Line Entry Specification (SMILES) of drugs and the amino acid sequences of protein targets.

**Conclusions:**

The effectiveness of the proposed approach was assessed through extensive experimentation on datasets pertaining to Enzymes, Ion Channels, and G-protein-coupled Receptors. The remarkable efficacy of DTIOG was showcased through the utilization of diverse similarity measures in order to calculate the similarities between drugs and targets. The combination of these factors, along with the incorporation of various classifiers, enabled the model to outperform existing algorithms in its ability to predict DTIs. The consistent observation of this advantage across all datasets underlines the robustness and accuracy of DTIOG in the domain of DTIs. Additionally, our case study suggests that the DTIOG can serve as a valuable tool for discovering new DTIs.

## Background

In recent years, pharmaceutical scientists have placed significant emphasis on both developing new drugs and repurposing existing ones based on established knowledge. Drug repurposing involves identifying new potential uses for existing drugs or discovering new drug candidates. An essential aspect of drug discovery and repurposing is identifying interactions between drugs and target proteins. These interactions can be determined through various methods, including in vivo, in vitro, and in silico experiments. Predicting DTIs can be broken down into four groups based on how much is known about the drug compounds and the target proteins: known drug versus known target, known drug versus new target candidate, new drug candidate versus known target, and new drug candidate versus new target candidate. The primary hypothesis of these studies is that if drug *d* interacts with protein *p*, then drug compounds similar to *d* are likely to interact with protein *p*, and proteins similar to *p* are likely to interact with drug *d*. Additionally, drug compounds similar to *d* are likely to interact with proteins similar to *p*. This idea supports the assumption that drugs and targets with similar traits may interact in similar ways. It shows how important it is to use similarity information to guess how drugs and targets might interact.

It has been known for a long time that experimental methods based on clinical trials are slow, expensive, and require a lot of resources to be used [1–4]. To mitigate these challenges, in-silico experiments have gained popularity as a cost-effective alternative. In-silico prediction of unknown DTIs has become a widely adopted approach for drug repurposing and development. This plan includes two main ways to guess how drugs will interact with their targets: molecular docking methods [5] and ML methods [6]. Researchers can quickly look into and guess how drugs might interact with their targets by using computer simulations and advanced ML algorithms. This speeds up the process of finding new drugs and makes drug development more targeted and effective.

For instance, in the context of COVID-19, in the beginning, because of the lack of specific effective antiviral therapies, a wide variety of strategies have been investigated to fight this pandemic’s [7]. Among them, one potential strategy is to inhibit the interaction between the virus and ACE2 receptors in host cells. In addition, various pharmaceuticals, such as glucocorticoids, COX inhibitors, and immunosuppressants, have been shown to effectively address the inflammatory response. Notably, mucolytic drugs may also mitigate pulmonary edema and combat viral infections. On the other hand, DL has emerged as a critical tool in the fight against the COVID-19 pandemic, offering valuable insights into epidemiology, diagnosis, and disease progression [8]. Some researchers are looking at the 3D or 2D structure of the SARS-CoV-2 virus proteins to learn more about drugs and compounds that could be used as drugs [9, 10]. However, the 3D structure of many targets or proteins is not known, which could make it harder for structure-based methods to make predictions. In some other approaches, the PPI networks have been studied to discover HP-PPI between SARS-CoV-2 and human proteins [11]. Other methods, such as SANE [12], try to find new uses for drugs that are already in use against COVID-19. The process involves integrating information pertaining to the drugs and the virus sequence into a framework designed for attention-based pre-search network embedding. Initially, the researchers gathered fundamental data pertaining to drug SMILES and virus sequences, alongside a valuable dataset encompassing drug-virus interactions known as the Human Drug-Virus Interaction Database (HDVD). The process of extracting sequence features relies on a configuration consisting of an encoder and a decoder.

On the other side, the problem of no verified virus-drug associations poses a significant challenge in the search for effective treatments against emerging viruses like SARS-CoV-2 [13]. However, the CMNMF technique offers a promising solution. By integrating multiple sources of biological data, including genetic information and protein interactions, CMNMF enables a holistic analysis of potential drug interactions. Unlike traditional methods, CMNMF doesn’t rely solely on existing associations, allowing it to navigate the cold-start problem efficiently. This approach not only accelerates the identification of potential drug candidates but also enhances the accuracy of predictions.

A KG serves as a structured data framework connecting entities and relationships. In this setting, KGE techniques turn entities and relationships into continuous vector spaces, which makes ML applications easier to use. This innovation has found practical use in the field of drug discovery, where applicable KGs have been increasingly created [14, 15]. These specialized KGs incorporate drugs, genes, and diseases as entities, capturing their intricate interactions as relationships. This integration of KGs and embedding techniques empowers researchers to gain valuable insights and make informed decisions in the complex landscape of drug development. Predicting the missing links between these entities can be viewed as one of several essential drug discovery tasks. In contrast, target discovery identifies missing links between genes and diseases. KGE models, which learn the low-dimensional representation of entities and relationships, have been employed to complete these tasks. These models are distinctive in that their predictions are components of processes that can culminate in physical experimentation in the real world and even clinical trials. The latter can incur substantial financial, regulatory, and time-related costs and significantly impact efforts to improve patient health. Recently, neural network-based approaches have become popular for KGE due to their ability to capture complex and non-linear relationships within the graph data. As an illustration, KGNN [16] takes a different approach by incorporating GCNs with neighborhood sampling, enabling the explicit extraction of neighborhood relations. SumGNN [17] utilizes KG to extract manageable pathways by incorporating a graph summarization module focused on subgraphs. Meanwhile, DDKG [18] refines drug embeddings by considering both neighboring node embeddings and triple facts through an attention mechanism. Lastly, KG2ECapsule [19] represents a noteworthy advancement by integrating capsule networks to model multi-relational DDI data explicitly based on biomedical KG in an end-to-end manner.

Although there are several methods for the prediction of DTIs that have shown promising results [20], some challenges remain. Current methods do not explicitly consider the drug–target knowledge graph (i.e., structured knowledge) and sequence data to make accurate predictions.

The contributions of this paper are summarized as follows:

1. The proposed approach uses the KG to generate the similarities between drugs–drugs and targets–targets and the KGEs of drugs and targets. We rely on the knowledge graph since it is a structured knowledge database in which entities (e.g., drugs or proteins) are represented as nodes and relationships between these entities are represented as edges, providing comprehensive and rich semantics for organizing and understanding information.
2. To capture the contextual information from other different embedding strategies, we use a KGE for drugs and ProtBERT embeddings [21] for target sequences. ProtBERT [21] is a variant of BERT specifically designed for protein sequences. ProtBERT embeddings are advantageous because they capture the intricate relationships between amino acids in proteins, including spatial and sequential dependencies. This representation captures contextual information, allowing the model to understand the sequential nature of amino acids in the protein. These embeddings are especially useful when dealing with tasks related to protein structure, function, and interactions. By combining these embeddings and feeding them into a specific classifier, we enable the model to understand both the semantic relationships between drugs and proteins in the knowledge graph and the sequential information within protein sequences. This combined representation provides a comprehensive view of the drug-protein interactions, allowing our model to make more informed predictions based on both structured knowledge and sequence data.
3. The approach can extract the embedding by focusing on the local representations from the SMILES of each drug and the amino acid sequence information of the protein target.

DTIOG extracts the characteristics of KG to better utilize the characteristics of the drug–target relationship. Predicting links with knowledge graph integration models requires the data to be modeled as a graph [22]. The goal is to predict new links between entities in the graph. We use bipartite graphs from biomedical knowledge bases to generate informative graphs around DTIs.

## Related work

Traditional computing methods for discovering DTIs can be broadly divided into two categories: ligand-based approaches and structure-based approaches. Docking simulations are used in structure-based approaches [23, 24], but they cannot always be used when the 3D structures of the target protein are not available. On the other hand, ligand-based techniques are another approach to discovering DTIs. Still, they must improve their accuracy when only a few binding ligands are available for the target protein [25]. Recently, there has been a lot of activity in bioinformatics using data-driven approaches, mainly ML and DL algorithms, to guess how biomolecules will connect with each other [26, 27]. These advanced techniques have become increasingly popular due to their ability to analyze complex biological data. Concurrently, network representation learning methods have emerged as a vital component in this endeavor. These methods can be broadly classified into three categories: matrix factorization-based, random walk-based, and neural network (NN)-based methods [28].

Unlike homogeneous networks, heterogeneous networks integrate data from various sources, such as drugs, targets, and related diseases. Several computational approaches have been proposed to fuse heterogeneous network data. For example, DeepWalk is a DL method that calculates similarities in a tripartite, heterogeneous network constructed from linked biomedical datasets [29]. The deepDTnet method [30] uses deep neural networks for graph representation algorithms to learn low-dimensional vector representations for drugs and targets/proteins that are still useful. This approach applies PU-matrix completion to predict new DTIs. The DTINet method [3] uses matrix factorization and graph embedding to guess new DTIs from a complex graph. Moreover, DTINet combines various types of drugs and target proteins to build a comprehensive, heterogeneous network.

AOPEDF [31] presents a computational approach for molecular target identification and drug repurposing centered around known drugs and targets. The first step of the method is to get reduced-dimensional vector representations of characteristics that capture arbitrary-order proximity from a biological network that links drugs, targets (i.e., proteins), and diseases. This network is highly connected and has many different types of connections. Subsequently, AOPEDF utilizes these informative vector representations for drugs and targets/proteins, employing a sequence of deep forest classifiers to deduce new DTIs.

Zhao et al. suggested the LGDTI method in [32]. It is a new way to determine DTIs by learning from large graph representations. This method gathers both local and global structural data about the graph, and it uses the GCNs to put together the node’s first-order neighbor data. Moreover, it learns the high-order neighbor information of nodes through the graph embedding method, DeepWalk. The resulting features are fed into a Random Forest classifier to infer new DTIs.

Cheng et al. [33] came up with the GraphMS model, which is an end-to-end network model made just for figuring out DTIs using low-level representations. One important thing about it is that it puts a lot of weight on node-level representation accountability. This is done by making sure that node-level and graph-level representations share as much information as possible with each other. GraphMS also keeps substructure information in the graph-level representation by making the information that flows between the graph-level and substructure representations better. Finally, the model learns meaningful feature embeddings from variant information using an autoencoder. This lets the model make DTI predictions that are accurate and reliable.

A computer method called DTiGEMS+ [34] combines graph embedding, graph mining, and putting together similarities from different sources of information. This method effectively combines similarity-based and feature-based approaches. It treats the problem of finding new DTIs as a link prediction problem in a complex network. By adding drug–drug and target–target similarity networks to the established DTIs graph, DTiGEMS+ creates this diverse network.

To infer new DTIs, the LRSpNM framework [35] utilizes a matrix completion technique, specifically minimizing the Laplacian regularized Schatten p-norm, to predict new DTIs. The method assumes that similar drugs interact with similar targets and vice versa, leading to a low-rank structure in the DTI matrix. Matrix completion algorithms can then efficiently approximate lower-rank matrices consistent with known interactions, aiding in discovering new DTIs. Schatten’s p-norm approximates the matrix rank, and the regularized Laplacian term is incorporated to enhance the prediction process. Additionally, as a significant portion of the DTI matrix contains unknown interactions, a pre-filling step is employed to improve prediction accuracy.

LRSpNM involves three main steps. Firstly, a pre-processing step is conducted to estimate partial unknown interaction probabilities by considering the *K* nearest neighbor profiles. Next, Laplacian matrices are computed for drugs and targets based on the original similarity matrices.

The presence of missing interactions in the training set can negatively impact DTI prediction models, reducing accuracy. To solve this problem, WkNNIR [36] is suggested. It combines WkNN with interaction recovery to guess what will happen on the full interaction matrix. WkNNIR has the advantage of appropriately weighting the importance of drug and target similarities based on their local imbalance. In the initial phase, WkNNIR calculates the recovered interactions, which replace the original interactions during the prediction process. Based on the idea that similar drugs interact with similar targets and targets interact with similar drugs, interactions that are missing can be guessed by looking at interactions with drugs or targets that are close by.

ALADIN [37] is a localized approach for predicting DTIs. This methodology involves three steps: representation based on similarity, ensemble based on projection, and prediction of new drugs and targets. In the representation step, drug–drug similarities represent the drugs in the similarity space. Specifically, drug $d_i$ is represented by a vector capturing its chemical similarity to all other drugs, and similarly, targets can be represented based on their similarities with other targets. Drug–drug and target–target similarities are computed based on known interactions using the Jaccard similarity. This better representation looks at both how similar two drugs (or targets) are chemically (genetically) and how similar they are to each other in terms of how they interact with each other.

The DTI-HeNE [38] method takes as inputs a bipartite graph of DTIs and two homogeneous graphs of drug–drug and target–target interactions. The BINE algorithm is then used to turn the DTI bipartite graph into two vectors of drugs and targets that are embedded in each other. The SNF algorithm, on the other hand, turns the uniform graphs of drug–drug and target–target interactions into two similarity matrices, one for drug–drug interactions and the other for target–target interactions. This study uses a knowledge graph as input to generate two KGEs for drugs and targets using KGE methods. Additionally, two similarity matrices, drugs-drugs and targets-targets, are generated from this KG using KGE similarity.

The algorithm iGRLDTI [39] is a recent approach designed to predict DTIs by enhancing the discriminative representations of drugs and targets in a latent feature space. It achieves this by constructing a complex HBIN that integrates biological knowledge about drugs, protein targets, and their interactions. iGRLDTI employs a node-dependent local smoothing strategy, which determines the propagation depth for each biomolecule in HBIN. This adaptive approach mitigates over-smoothing issues and enhances the discriminative power of feature representations. Subsequently, a GBDT classifier is applied to predict novel drug–target interactions. Similarly to iGRLDTI, LG-DTI [40] operates over a heterogeneous information network, modeling the DTI network equipped with biological data on drugs and targets. LG-DTI utilizes both local and global representations of drugs and target proteins. Firstly, it learns local representations from drug molecular structures and protein sequences. Secondly, it employs a semi-supervised heterogeneous network embedding method to capture global representations, taking into account the topological structure of the DTI network. These local and global representations are combined using a concatenation aggregation function, forming the final representations of drugs and targets. These representations are then fed into a Random Forest classifier, enabling LG-DTI to predict DTIs effectively.

We proposed an innovative approach for predicting DTIs, leveraging both contextual and local strategies. In terms of contextual information, DTIOG utilizes KGE techniques such as the DistMult model [41] to generate drug and target embedding vectors. These vectors are derived from the knowledge graph, capturing associations and similarities between drugs and targets. Instead, DTIOG uses ProtBERT, a language model that has already been trained on protein sequences, to figure out how amino acids in proteins are put together. These embeddings, obtained through either KGE or ProtBERT, are integrated into the process of prediction. Adding to that, some recent deep learning-based models that have been pre-trained on a large corpus of protein sequences, such as ProtBERT, have been utilized to extract features of the proteins. For example, ProtBERT can be used to provide meaningful, context-aware representations of protein sequences, which are crucial for the accurate identification of lysine glutarylation sites [42].

For the local strategy, DTIOG gets information about drugs by using the RDKit [45] library to turn SMILES representations into molecular fingerprints. The Avalon fingerprint generator identifies specific fragments within the molecular structure, creating numerical representations for each drug. Regarding protein sequences, DTIOG processes them into feature vectors based on amino acid biochemical properties. A sliding window of size 3 categorizes amino acids into groups (i.e., non-polar, polar neutral, acidic, and basic), transforming sequences into numerical representations.

Additionally, DTIOG calculates the drug–drug and target–target similarity matrices from contextual or local embedding vectors by employing a variety of similarity metrics, including cosine similarity, Euclidean distance, Jaccard similarity, Manhattan distance, and Pearson correlation. These diverse metrics contribute to the creation of comprehensive similarity matrices, enabling a more nuanced understanding of the relationships between drugs and targets. By combining contextual embeddings from KGE or ProtBERT or combining local features derived from SMILES and protein sequences, DTIOG enables a comprehensive understanding of drug–target relationships. It is worth noting that strategies for combining drug and target embeddings are derived from the DTI-HeNE method [38]. These embeddings and features are fed into specific classifiers, allowing the model to grasp both the semantic associations in the knowledge graph and the sequential patterns within protein sequences, enhancing its accuracy in predicting DTIs.

## Materials and methods

### Contextual embedding

#### The KGE

The DTIOG uses a network-based approach to build a weighted heterogeneous graph from the DTI’s network. This is further enhanced by incorporating different drug and target similarity networks. The resulting graph, denoted as *G*(*V*, *E*), comprises a set of n drug nodes, $D = {d^1, d^2,\ldots , d^n}$, and a set of l target nodes, $T = {t^1, t^2,\ldots , t^l}$. Notably, the knowledge graph G constructed this way contains 131 types of edges. Given *G*, the DTIs prediction problem may be defined as a link prediction challenge, with the objective being to predict the unknown true interactions between drugs and targets/genes.

**Fig. 1:**
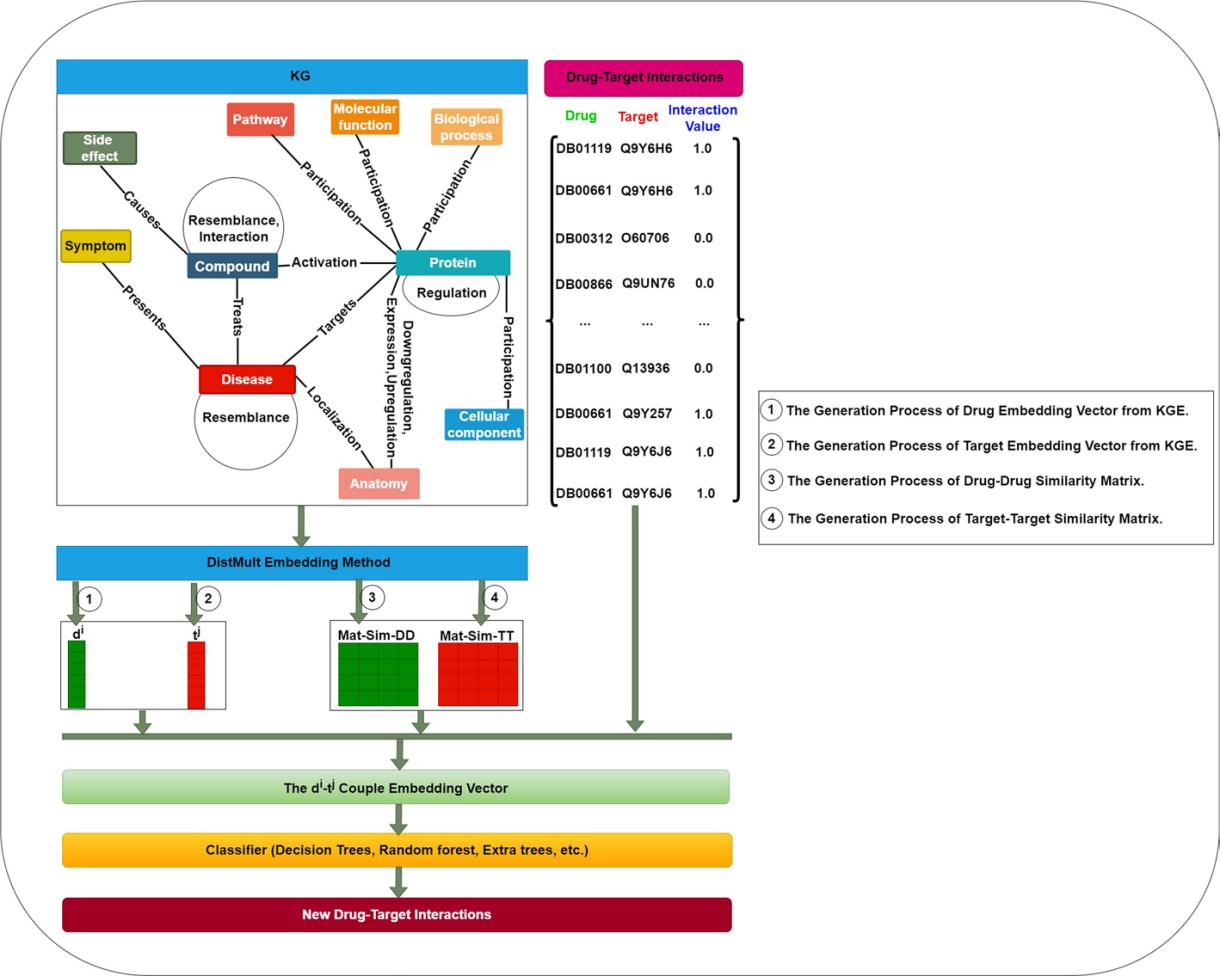
The schematic workflow of DTIOG

The KGE strategies will be implemented to introduce the features of each drug and target pair. Specifically, the DistMult embedding method will represent each node in the *KG* with a feature vector smaller than the actual number of nodes while preserving the graph’s structure and attributes. Then, after using the heterogeneous and complex graph *KG* to learn feature representations for each drug and target, different similarity metrics between each pair of drugs and each pair of targets will be calculated to create various similarity matrices (i.e., drug–drug and target–target similarities). The similarity matrices will be calculated as part of our method’s workflow. Then, by combining the association and similarity of drug and target matrices, multiple classifiers will be employed to deduce new DTIs (cf. Fig. 1). Our method combines different chemical, genomic, phenotypic, and cellular networks to make features that are useful for biology and pharmacology. Combining the informative vector representations of drugs and targets yields these features.

#### The ProtBERT embedding

The DTIOG algorithm can rely on ProtBERT embedding [21] of protein sequences to serve as an alternative to traditional KGE. The method uses ProtBERT, a cutting-edge pre-trained language model made just for protein sequences, to gather contextual information. This lets the model understand how the amino acids in the protein are put together. The ProtBERT model [21] is specific to uppercase amino acids and works with a MLM objective. It is trained using these amino acids, so it can only work with capital-letter amino acids. The ProtBERT model was initially trained on the UniRef100 [43] dataset, which encompasses a staggering 217 million protein sequences. It is possible to get a full picture of protein properties by using these embeddings to record complex sequence patterns and biological features.

### Local embedding

#### Drug feature extraction

To extract drug features, we retrieved the SMILES representations of each drug from DrugBank [44]. Subsequently, we utilized the RDKit [45] library to convert these SMILES strings into molecular fingerprints. For each drug, the positions within its fingerprint were assigned a value of 1 if a corresponding fragment was identified within the molecular structure and 0 otherwise. We opted for the Avalon fingerprint generator, which enumerates specific paths and feature classes within the molecular graph, to perform this conversion. We employed a dimensionality reduction strategy using autoencoders [46] to obtain a more compact representation of each drug, resulting in a dimensionality of 64.

#### Protein feature extraction

The DTIOG algorithm provides a systematic way to convert protein sequences into numerical representations (i.e., feature vectors) based on the biochemical properties of amino acids. The process begins by converting amino acid sequences to feature vectors with a sliding window of size 3. It categorizes amino acids into non-polar, polar-neutral, acidic, and basic groups. The algorithm reads input protein sequences from a FASTA downloaded from the UniProt database [47] and processes them to create feature vectors representing the sequence characteristics. Each feature vector is of length 64 and is built by considering overlapping triplets of amino acids in the protein sequences.

### The process of predicting novel DTIs

**Fig. 2:**
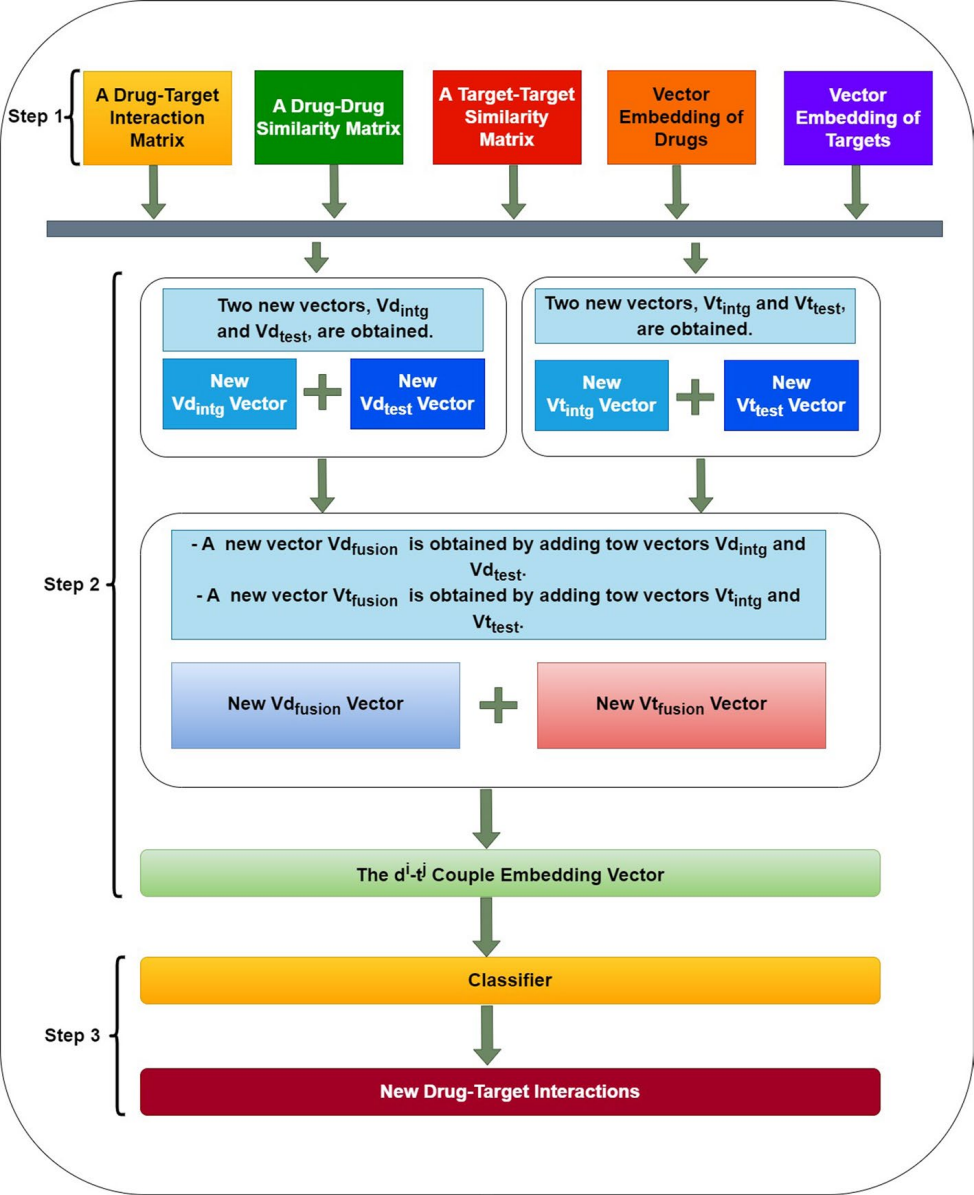
The process of predicting novel drug–target interactions

**Fig. 3:**
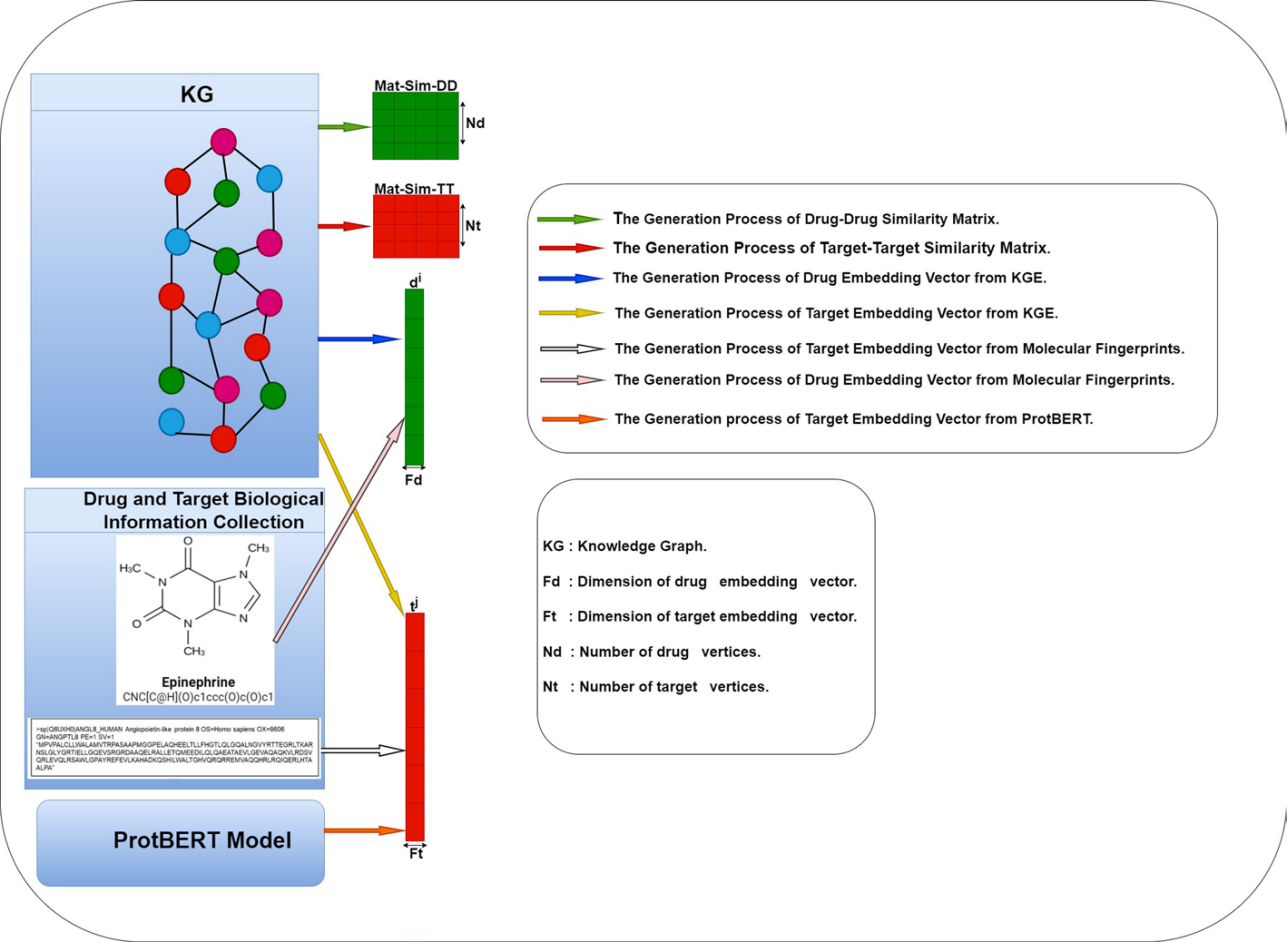
This flowchart illustrates the first two steps of DTIOG, which are the generation of two vector embeddings for drugs (i.e., $d^i$), targets (i.e., $t^j$), drug–drug similarity (i.e., $Mat-Sim-DD$) and target–target similarity (i.e., $Mat-Sim-TT$) generated from KGE using Cosine similarity by computing the DistMult model

**Fig. 4:**
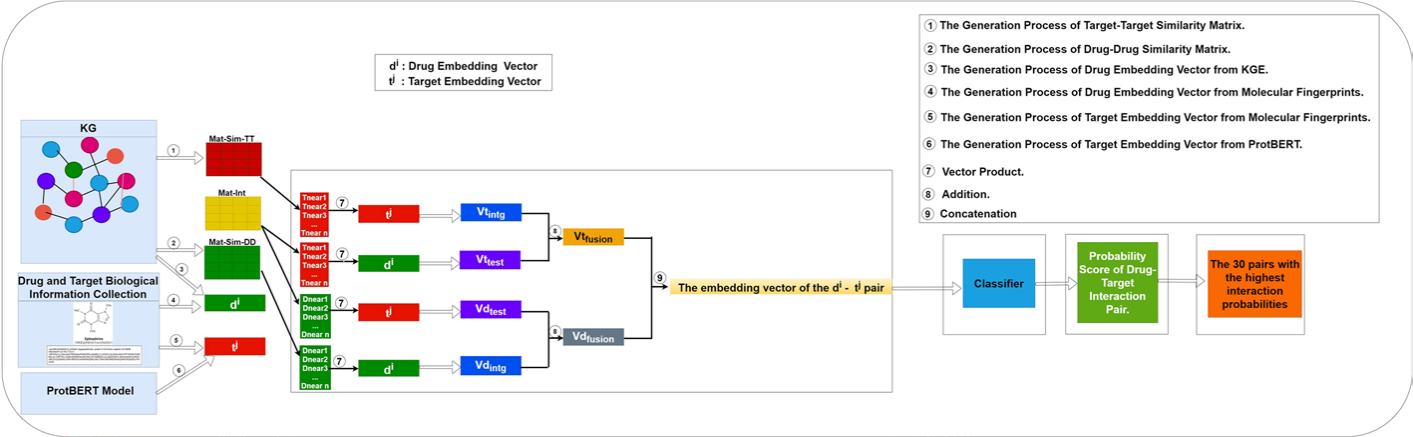
The process of predicting novel drug–target interactions by emphasizing the creation of an integrated embedding vector for the pair $d^i$ and $t^j$

This section starts with a description of the rationale behind the calculation of contextual and local embeddings during the forecasting phase. Then, a thorough explanation of the overall architecture follows the problem formulation explanation. Figure 2 presents the three main steps:

(i) *Step 1*: The input consists of a matrix representing DTIs, along with two vector embeddings for drugs and targets (cf. Fig. 3). Subsequently, DTIOG computes drug–drug and target–target similarity matrices in the prediction process. It’s essential to highlight that the similarity among drugs, proteins, and their embedding vectors is derived from three scenarios: (a) One way to get similarity and embedding between drugs and targets is to use KGE; (b) Another way is to get similarity and embedding between drugs using KGE and then compute ProtBERT embeddings for the targets; (c) Finally, we get similarity and embedding between drugs using their fingerprints. We also read the targets’ protein sequences in FASTA format and put amino acids into four groups (i.e., basic, acidic, non-polar, and polar neutral). We then process them to create feature vectors representing the sequence characteristics, with each feature vector having a length of 64.
(ii) *Step 2*: Begin by identifying the row corresponding to the drug ${d^i}$ in the drug–drug similarity matrix. Then, sort the values in this row from largest to smallest and select the drugs associated with the *n* largest values. Similarly, in the target–target similarity matrix, find the row corresponding to the target ${t^j}$. Then, identify the *n* targets with the highest similarity. Next, multiply the embedding vector of ${d^i}$ by the corresponding weights of the selected *n* nearest drugs in the drug–drug similarity matrix. Repeat this process for each selected drug, summing up the obtained products to create a new feature $Vd_{intg}$. Apply the same procedure to the embedding vector of ${t^j}$. Multiply it by the corresponding weights of the selected *n* nearest targets in the target–target similarity matrix and sum up these products to obtain a new feature $Vt_{intg}$. The primary objective at this step is to integrate the drug–drug and target–target similarity matrices into the respective embedding vectors ${d^i}$ and ${t^j}$. Additionally, multiply the embedding vector $t^j$ by the weights in the bipartite DTIs matrix (i.e., $Mat-int$) corresponding to the selected n nearest drugs and $t^j$ individually. Sum the products generated for each drug to obtain a new feature $Vd_{test}$. Simultaneously, multiply the embedding vector $d^i$ by the weights in the bipartite DTIs matrix (i.e., $Mat-int$) corresponding to the n selected nearest targets and $d^i$ individually. Sum up the products obtained for each target to create a new feature $Vt_{test}$. This step enables the modeling of interactive pathway information related to known interactions between drugs (more similar to $d^i$) and $t^j$, as well as known interactions between $d^i$ and targets (more similar to $t^j$). Subsequently, a new embedding vector, denoted as $Vd_{fusion}$, is computed by summing the vectors $Vd_{intg}$ and $Vd_{test}$. Similarly, a novel embedding vector, denoted as $Vt_{fusion}$, is calculated by summing the vectors $Vt_{intg}$ and $Vt_{test}$.
  Finally, concatenating the embedding vectors $Vd_{fusion}$ and $Vt_{fusion}$ enables the creation of an integrated embedding vector for the pair $d^i$ and $t^j$ (cf. Fig. 4). This process effectively incorporates features from both the bipartite DTIs network and the drug–drug and target–target similarity matrix, fostering a comprehensive representation of the relationships between drugs and targets. It is worth noting that the functionalities of *Step 2* are the same strategies used by the DTI-HeNE approach [38] to concatenate the embedding vectors $Vd_{fusion}$ and $Vt_{fusion}$.
(iii) *Step 3*: The feature vector is represented by $X = {x_1, x_2, \ldots , x_{n*l}}$ and their labels $Y= {y_1, y_2, \ldots , y_{n*l}}$ where $n*l$ corresponds to the number of drugs multiplied by the number of targets, which constitutes the number of all possible drug and target pairs. Therefore, if there is a known interaction for the drug–target pair, the class label *y* for this pair equals 1 ($y = 1$); otherwise, the class label is equal to zero ($y = 0$). Thus, it is a binary classification task. The aim is to find novel DTIs with high accuracy and a low false-positive rate. The negative samples in our approach are generated by augmenting a bipartite graph with information regarding the interactions between drugs and targets. Subsequently, it generates lists to track these interactions and calculates the overall number of potential drug–target pairs. Afterwards, it detects and eliminates interactions that are already familiar from the dataset. These remaining negative examples act as substitutes for interactions that have not been recorded in the original dataset, indicating a deficiency of information regarding them. Simultaneously, the code calculates the probability of these potential interactions, a pivotal stage in forecasting interactions that we have not previously encountered. Ultimately, a specific classifier is employed to forecast novel DTIs.

### Generation of embedding vectors for drugs ($d^i$) and targets ($t^j$)

#### Contextual embedding vectors

The input is a bipartite graph of DTIs, and two embedding vectors, $d^i$ and $t^j$, have been generated in this step (cf. Fig. 3). DTIOG will use one strategy from the contextual or local categories to develop the two embedding vectors of drugs $d^i$ and targets $t^j$. In the context of a KG [48] consisting of triplets representing facts $\omega = {\le h, r, t \ge }$ and a fixed embedding space of dimension *d*, KG embedding aims to map each entity $h \in E$ and relation $r \in R$ into a continuous vector in a uniform embedding space of dimension $k=d$. This transformation turns the KG into a set of vectors, capturing its information and enabling computations on the graph. KGE tries to make a small, low-dimensional feature space that keeps important structural and property data about the graph. This makes it easier to do calculations with entities and relations. In the field of KGE, techniques can be broadly classified into two categories: (1) representation learning models centered on triplet facts [49] and (2) representation learning models based on entity descriptions [50]. The DTIOG approach specifically emphasizes the use of triplet fact-based representation learning models, particularly the DistMult variant.

When it comes to protein sequences that are stored as text, ProtBERT uses transformer-based language models, more specifically BERT [51]. Given a set of protein sequences denoted as $\text {Proteins} = {p_1, p_2, \ldots , p_n}$, ProtBERT learns to embed these sequences into continuous vector representations. The complex dependencies and relationships between amino acids in proteins are captured by ProtBERT by processing them as sequences of amino acids. This helps it understand the subtleties of protein sequences.

#### Local embedding vectors

Molecular fingerprinting is a technique used to represent chemical structures numerically. Initially, the chemical structures of drugs are encoded into SMILES representations ($\text {SMILES}_i$). These SMILES strings are then transformed into binary molecular fingerprints ($F_i$), where each element ($f_{ij}$) in the fingerprint vector indicates the presence (1) or absence (0) of specific predefined chemical substructures within the molecule. The Avalon fingerprint generator is employed to identify these substructures. Subsequently, a dimensionality reduction process using autoencoders ($F_i^{\prime } = \text {Autoencoder}(F_i)$) is applied to obtain a compact representation of the fingerprint, reducing its dimensionality to, for example, $d = 64$. This reduced-dimensional fingerprint ($F_i^{\prime }$) serves as a concise numerical descriptor of the drug’s chemical composition, enabling efficient computations and analysis in drug discovery and related fields.

In the realm of protein sequence analysis, the goal is to transform amino acid sequences into numerical representations, often referred to as feature vectors, which can be utilized for various computational tasks. Let us consider a protein sequence $P = \{a_1, a_2, \ldots , a_n\}$, where $a_i$ represents the (*i*th) amino acid in the sequence. Each amino acid $a_i$ can be associated with specific biochemical properties, denoted as $\phi (a_i) = \{p_1, p_2, \ldots , p_m\}$, where $p_j$ represents the (*j*th) biochemical property of the amino acid.

The DTIOG algorithm employs a systematic approach to convert protein sequences into feature vectors. This process begins by adopting a sliding window of size $w$ (in this case, $w = 3$) to capture local structural information. For each window position $i$, a subsequence $S_i = \{a_i, a_{i+1}, \ldots , a_{i+w-1}\}$ is extracted. Subsequently, each amino acid in the subsequence is mapped to its corresponding biochemical properties using the function $\phi (\cdot )$. These biochemical property vectors are then concatenated to form a composite feature vector $V_i$ for the window $S_i$:

$$\begin{aligned} V_i = [\phi (a_i), \phi (a_{i+1}), \ldots , \phi (a_{i+w-1})] \end{aligned}$$

As a result, each $V_i$ captures the local biochemical characteristics of the corresponding window in the protein sequence. Considering a protein sequence of length $n$, this process generates $n-w+1$ feature vectors.

Moreover, the amino acids can be categorized into distinct groups based on their properties. Let $A = \{a_1, a_2, \ldots , a_n\}$ represent the set of amino acids in the protein sequence, and $G = \{g_1, g_2, \ldots , g_k\}$ denote the set of biochemical groups. The algorithm categorizes amino acids into these groups, allowing for the incorporation of group-level information into the feature vectors. Consequently, the feature vectors $V_i$ are enhanced with group-level biochemical properties, providing a comprehensive representation of the protein sequence in a numerical format.

### Generation of new drug–target pair embedding vectors

During this particular phase, we utilize the two embedding vectors (i.e., $\overrightarrow{d^i}$ and $\overrightarrow{t^j}$), along with the two matrices of drug–drug similarity and a target–target similarity (i.e., $Mat-Sim-DD$ and $Mat-Sim-TT$) and the bipartite DTIs matrix (i.e., $Mat-int$) to create a new embedding vector of the pair $d^i$ and $t^j$.

By a specific process [38], we first obtain $Vd_{intg}$ and $Vt_{intg}$. Next, $Vd_{test}$ and $Vt_{test}$ are generated. Then, $Vd_{intg}$ and $Vd_{test}$ are added to obtain $Vd_{fusion}$, and $Vt_{intg}$ and $Vt_{test}$ are added to obtain $Vt_{fusion}$. Finally, $Vd_{fusion}$ and $Vt_{fusion}$ are concatenated to obtain an embedding vector of the pair $d^i$ and $t^j$.

### Prediction of DTIs

After getting an embedding vector of the pair $d^i$ and $t^j$, different classifiers are used to give each pair’s interactions in the vector a probability. Subsequently, the likelihood of a possible interaction between each drug–target pair is estimated. The different classifiers provide us with the probability of interaction for each couple, and we have chosen to display the couples with the highest probabilities (e.g., the top 10 couples). By showing the 10 pairs with the highest interaction probabilities, we can prioritize pairs more likely to have favorable interactions [38]. This approach lets us focus on the most reliable and significant predictions while filtering out less relevant results. Consequently, we can dedicate our efforts to thoroughly analyzing the most pertinent interactions. To determine the pair interaction probabilities $d^i$ and $t^j$, we employed nine distinct classifiers, including the RF, DT, MLP, K-Neighbors Classifier, Bagging Classifier, Gradient Boosting Classifier, GaussianNB, SGD, etc.

### Problem formalization

DTIOG pseudo-code is given in Algorithm 1. The approach takes as inputs the DTIs matrix (i.e., $Mat-int$), the embedding vectors $d^i$, and $t^j$. Initially, we compute the embedding similarity between the set of drugs and the set of targets by utilizing the predefined cosine similarity function. This step results in the generation of similarity matrices for drugs (i.e., $Mat-Sim-DD$) and for targets (i.e., $Mat-Sim-TT$). Additionally, alternative predefined functions, such as Euclidean distance, Jaccard similarity, Manhattan distance, or Pearson correlation, can be invoked. Subsequently, we calculate the dot product of the drug embedding vector $d^i$ and the weights $w^z_{d}$ of the *n* closest drugs $D^{near}$ in the $Mat-Sim-DD$ to obtain $Vd_{intg}$. Adding to that, we computed the dot product of the target embedding vector $t^j$ and the weights $w^z_{t}$ of the *n* closest targets $T^{near}$ in the $Mat-Sim-TT$ to obtain $Vt_{intg}$. $Vd_{intg}$ and $Vt_{intg}$ are computed using the following equations [38]:

1 $$\begin{aligned}{} & {} Vd_{intg} = \sum _{d^z \in D^{near}} W_d^z \overrightarrow{d^i} \end{aligned}$$

2 $$\begin{aligned}{} & {} Vt_{intg} = \sum _{t^z \in T^{near}} W_t^z \overrightarrow{t^j} \end{aligned}$$

Then, we compute the dot product of the target embedding vector $t^j$ by the weights $w^z_{d^i}$ of the *n* closest drugs $D^{near}$ in the $Mat-int$ to obtain $Vd_{test}$. Therefore, we computed the dot product of the drug embedding vector $d^i$ by the weights $w^z_{t^j}$ of the *n* closest targets $T^{near}$ in the $Mat-int$ to obtain $Vt_{test}$. $Vd_{test}$ and $Vt_{test}$ are computed using the following equations [38]:

3 $$\begin{aligned}{} & {} Vd_{test} = \sum _{d^z \in D^{near}} W^z_{t^j} \overrightarrow{t^j} \end{aligned}$$

4 $$\begin{aligned}{} & {} Vt_{test} = \sum _{t^z \in T^{near}} w^z_{d^i} \overrightarrow{d^i} \end{aligned}$$

Then $Vd_{intg}$ and $Vd_{test}$ were added to obtain $Vd_{fusion}$. On the other side, $Vt_{intg}$ and $Vt_{test}$ were added to obtain $Vt_{fusion}$. $Vd_{fusion}$ and $Vt_{fusion}$ are computed using the following equations [38]:

5 $$\begin{aligned}{} & {} Vd_{fusion} = Vd_{intg} + Vd_{test} \end{aligned}$$

6 $$\begin{aligned}{} & {} Vt_{fusion} = Vt_{intg} + Vt_{test} \end{aligned}$$

Meanwhile, the two vectors $Vd_{fusion}$ and $Vt_{fusion}$ have been concatenated to obtain the new embedding vector of the pair of $d^i-t^j$ [38]. Finally, we use a certain classifier to predict new DTIs based on a fresh embedding vector of $d^i$ and $t^j$.

**Algorithm 1:**
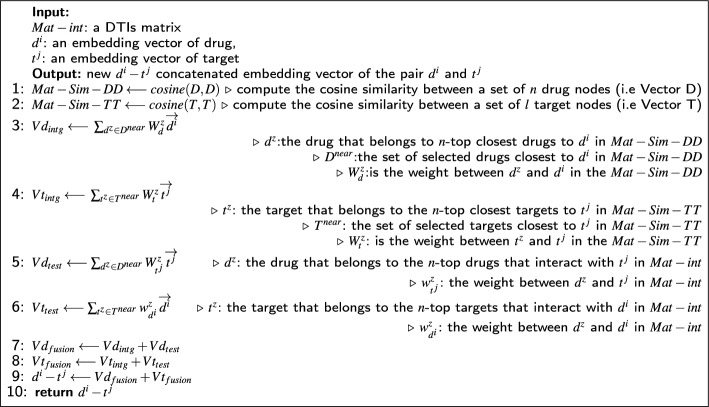
The process of concatenating the embedding vector of the pair *d*^*i*^ and *t*^*j*^

### Illustrative case

**Fig. 5:**
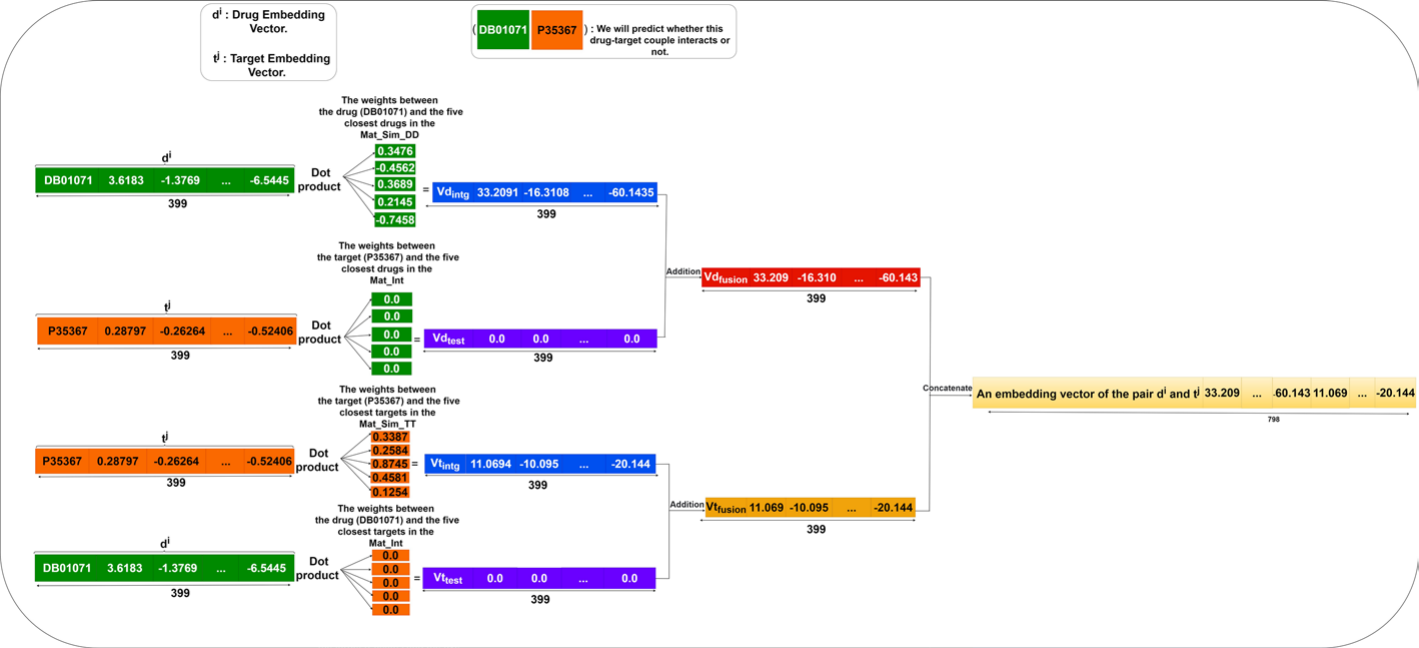
A toy example representing the different steps to obtain new pair vector $d^i-t^j$

Figure 5 presents an illustration that outlines the process of obtaining the vector for the pair $d^i-t^j$. The pair (*DB*01071, *P*35367) and its respective embedding vectors were utilized to execute several steps in generating new vectors for drug–target pairs:

Firstly, we calculate the dot product of the drug embedding vector $d^i$ associated with DB01071 and the top-5 closest drugs in $Mat-Sim-DD$ to acquire $Vd_{intg}$. Next, we perform the dot product of the target embedding vector $t^j$ associated with P35367 with the weights of the top-5 closest drugs in $Mat-int$ to obtain $Vd_{test}$. Subsequently, $Vd_{intg}$ and $Vd_{test}$ are added to yield $Vd_{fusion}$.

Secondly, we compute the dot product of the target embedding vector $t^j$ associated with P35367 and the five closest targets in $Mat-Sim-TT$ to obtain $Vt_{intg}$. Similarly, we compute the dot product of the drug embedding vector $d^i$ associated with DB01071 with the weights of the five closest targets in $Mat-int$ to obtain $Vt_{test}$. Then, $Vt_{intg}$ and $Vt_{test}$ are added to produce $Vt_{fusion}$. As a result, both vectors $Vd_{fusion}$ and $Vt_{fusion}$ are concatenated to form the new embedding vector of the pair $d^i$ and $t^j$.

Finally, DTIOG employs a selected classifier to predict new DTIs based on the newly created embedding vector of the pair $d^i$ and $t^j$.

## Results and discussion

### The input data

**Fig. 6:**
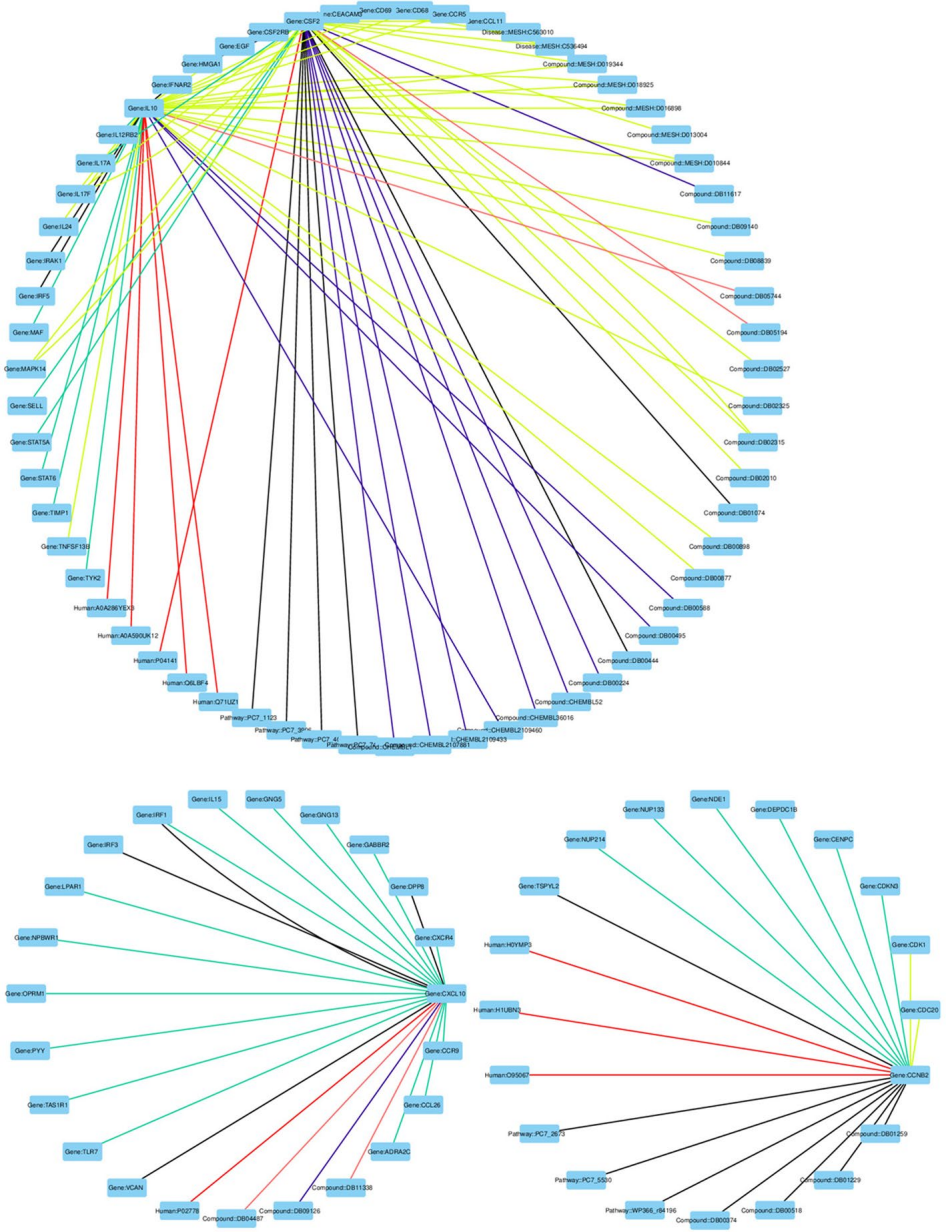
An example of the built knowledge graph by DTIOG that includes roughly 97 edges across 21 types of relationships connecting drugs, diseases, proteins, genes, PPIs for the gene *IL10, CSF2, CXCL10*, and so on the relation used in this example are from different sources, namely **a** the *DGIDB* [52] database (blue color); **b** the DrugBank database (purple color); **c** the GNBR [53] database (yellow color); **d** Hetionet biomedical knowledge (black color); **e** the STRING database (green color); and **f** UniProt database (red color)  (color figure online)

**Fig. 7:**
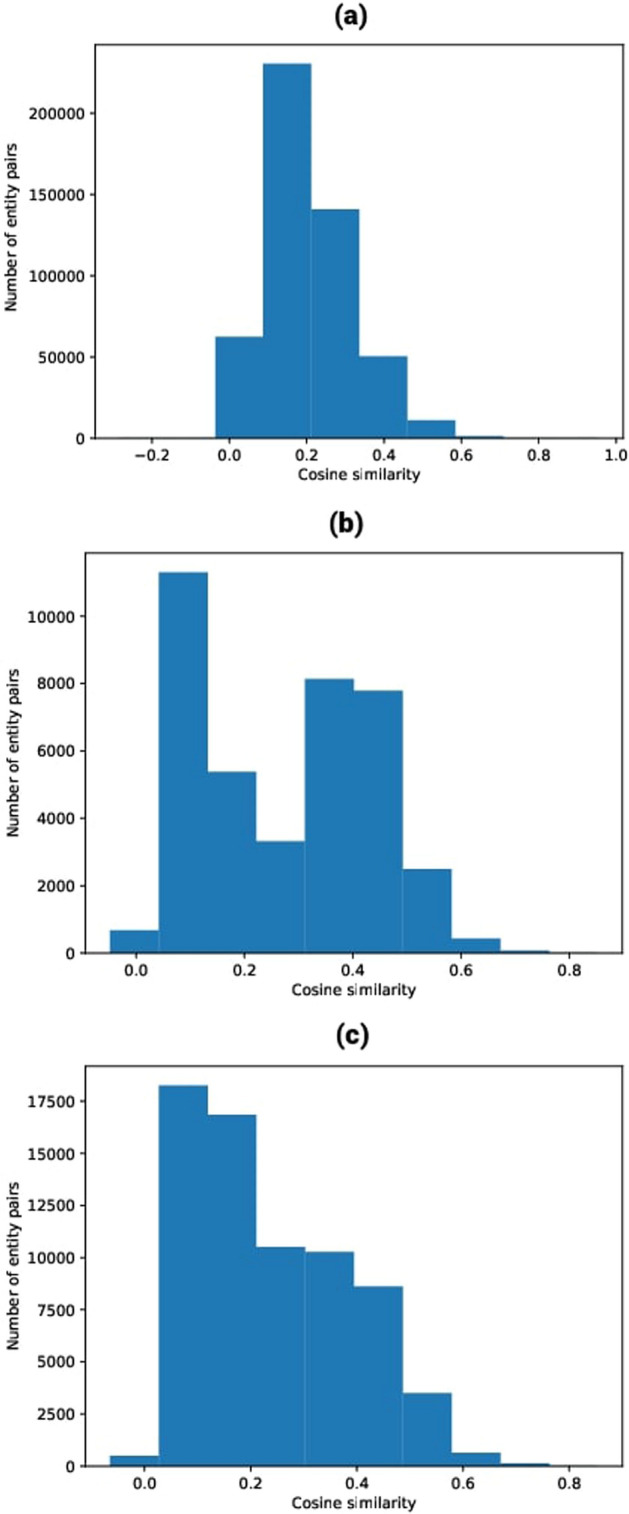
Entity cosine similarity distribution for: **a** ENZ data, **b** GPCR data, **c** IC data

Using the DGLKE methodology, we integrate various chemicals, genomic, phenotypic, and cellular networks to generate meaningful feature representations for drugs and targets. By combining informative vector representations, we construct a comprehensive knowledge graph comprising approximately 29 million edges that span 131 relationships connecting drugs, diseases, proteins, genes, PPIs for SARS-CoV-2, homo-sapiens, side effects, and more. Table 1 showcases 20 well-established relationships established during the network’s development. In Fig. 6, we present a subset of the constructed KG that interconnects drugs, genes, and PPIs for certain genes. Notably, we emphasize the sources of some connections used in this illustration, which were derived from diverse repositories, including the DGIDB [52] database, the DrugBank database (version 5.1.8), the GNBR [53] database, Hetionet (version 1.0) [54] biomedical knowledge, the STRING [55] database (version 11.0), and the UniProt [47] database. After that, the *DistMult* model has been used to learn the representations of the entities and relationships in an informative, low-dimensional vector space. We choose the *DistMult* [41] than using the TransE [56], TransR [57] since it yields the best evaluation of the training model in terms of *MRR*, *Hits*@1, *Hits*@3 and *Hits*@10 (cf. Table 2) and in terms of entity cosine similarity distribution (cf. Fig. 7). The evaluation experiments of these methods are carried out using a set of reference datasets. Specifically, this dataset is used by [58], which is typically used in DTI prediction, targeting ENZ, IC, and the GPCR. It consists of three different data subsets:

1. ENZ consists of 346 drugs, 657 proteins and 2926 interactions.
2. IC comprises 169 drugs, 204 proteins and 1476 interactions.
3. GPCR encompasses 188 drugs, 94 proteins and 634 interactions.

During the prediction process and for the embedding sizes of the drugs and proteins, we fixed the following: (a) 400 dimensions for the prediction process based on KGE for the ENZ, GPCR, and IC datasets; (b) 150 dimensions for the prediction process based on KGE and ProtBERT for the IC dataset; (c) 90 dimensions for the prediction process based on KGE and ProtBERT for the GPCR dataset; (d) 64 dimensions for the prediction process based on local strategies (i.e., molecular fingerprint and protein characteristics) for the ENZ, GPCR, and IC datasets.

**Table 1:** 20 known relationships in the knowledge graph network

Relationship	Species	Database	Total interactions
Gene-disease	SARS-CoV-2	CTD database [61]	28
Gene-drug	Homo-sapiens	CTD database [61]	9291
Protein–drug	Homo-sapiens	DrugBank [44]	20, 992
Protein–protein	Homo-sapiens	STRING database [55]	11,606735
Protein–protein	Mus-musculus	STRING database [55]	10, 016742
Protein-GOT CC	Mus-musculus	UniProt-GOA [62]	69, 373
Protein-GOT BP	Mus-musculus	UniProt-GOA [62]	125, 658
Protein-GOT MF	Mus-musculus	UniProt-GOA [62]	56, 322
Protein-GOT CC	Homo-sapiens	UniProt-GOA [62]	65, 295
Protein-GOT BP	Homo-sapiens	UniProt-GOA [62]	101, 177
Protein-GOT MF	Homo-sapiens	UniProt-GOA [62]	54, 638
Protein-gene	SARS-CoV-2	UniProt [47]	18, 387
Protein-gene	Homo-sapiens	UniProt [47]	195, 659
Protein-gene	Mus-musculus	UniProt [47]	110, 421
Anatomy-gene	Homo-sapiens	Hetionet [54]	726, 495
Gene-pathway	Homo-sapiens	Hetionet [54]	84, 372
Drug-side effect	Homo-sapiens	Hetionet [54]	138, 944
Drug-gene	Homo-sapiens	GNBR [53]	80, 803
		DrugBank [44]	24, 801
		IntAct [63]	1, 805
		DGIdb [52]	26, 290
		Hetionet [54]	51, 429
		Bibliography	25, 666
Disease-gene	Homo-sapiens	GNBR [53]	95, 399
		Hetionet [54]	27, 977
		Bibliography	461
Drug-disease	Homo-sapiens	Drugbank [44]	4, 968
		GNBR [53]	77, 782
		Hetionet [54]	1, 145

**Table 2:** Test average evaluation of the training of the DistMult model

Metrics	Value
MRR	0.527
Hits@1	0.399
Hits@3	0.594
Hits@10	0.785
Time	3008,041 s

It is worth noting that we compute the PCA [59] in order to reduce the dimensions of each vector, enabling them to have the same shape as the drug and target embedding vectors.

### Experimental setup and evaluation metrics

We have applied DTIOG in 9 different modes: DTIOG-RF, DTIOG-DT, DTIOG-MLP, DTIOG-KNeighbors, DTIOG-BaggingClassifier, DTIOG-GradientBoosting, DTIOG-GaussianNB, DTIOG-SGD, and DTIOG-ETC, utilizing the classifiers RF, DT, MLP, K-Neighbors, Bagging Classifier, Gradient Boosting, GaussianNB, SGD, and ETC, respectively. We compared DTIOG versus the pioneering approaches of the literature, namely iGRLDTI [39], DTI-HeNE [38], BLM-NII [60], and ALADIN [37]. It is worth noting that the number of epochs is 10, used by all the DTIOG variants and all their competitors. Table 3 provides an overview of the parameters of the classifiers used in our comparisons. Similar to the DTIOG strategy, the iGRLDTI method involves obtaining drug information by utilizing the RDKit library [45] by converting SMILES representations into molecular fingerprints, and we specifically use the Avalon fingerprint generator to identify distinct fragments in the molecular structure. In the case of protein sequences, we transform them into feature vectors based on the biochemical properties of amino acids. Using a sliding window of size 3, we categorize amino acids into groups such as non-polar, polar neutral, acidic, and basic, thereby converting the sequences into numerical representations.

**Table 3:** The classifier parameters are fixed by the choice from three scenarios responsible for determining the similarity between drugs, proteins, and their embedding vectors

Classifiers	KGE	KGE-ProtBERT	Molecular fingerprint and protein characteristics
ETC	n-estimators = trees, random-state = 1357	n-estimators = trees, random-state = 1357	n-estimators = trees, random-state = 1357
DT	random-state = 1357	random-state = 1357	random-state = 1357
MLP	solver = lbfgs, alpha = 1e−5, hidden-layer-sizes = (5, 2), random-state = 1	solver = lbfgs, alpha = 1e−5, hidden-layer-sizes = (240, 96), random-state = 1	solver = lbfgs, alpha = 1e−5, hidden-layer-sizes = (240, 96), random-state = 1
SGD	loss = log, penalty = l2, max-iter = 5	loss = log, penalty = l2, max-iter = 2	loss = log, penalty = l2, max-iter = 2
Gaussian-NB			
Gradient Boosting	n-estimators = 100, learning-rate = 1.0,max-depth = 1, random-state = 0	n-estimators = 100, learning-rate = 1.0,max-depth = 2, random-state = 0	n-estimators = 100, learning-rate = 1.0,max-depth = 2, random-state = 0
Bagging Classifier	KNeighborsClassifier(), max-samples = 0.5, max-features = 0.5	KNeighborsClassifier(n-neighbors = 1),max-samples = 1, max-features = 1	KNeighborsClassifier(n-neighbors = 1),max-samples = 1, max-features = 1
K-Neighbors	n-neighbors = 7	n-neighbors = 2	n-neighbors = 2
RF	n-estimators = trees, n-jobs = 6, criterion = c, class-weight = balanced, random-state = 1357	n-estimators = trees, n-jobs = 6, criterion = c, class-weight = balanced, random-state = 1357	n-estimators = trees, n-jobs = 6, criterion = c, class-weight = balanced, random-state = 1357

We employ a diverse set of evaluation metrics to provide a holistic assessment of our approach for drug–target interaction prediction. Using a combination of metrics, including AUC, AUPR, ACC, MCC, and the F1, allows us to examine the performance of the model from various angles. These metrics collectively offer insights into different aspects of the prediction, such as the ability to distinguish between positive and negative instances, precision in correctly identifying true positives, and the model’s balance between sensitivity and specificity. By employing this array of metrics, we ensure a well-rounded evaluation that takes into account the intricacies of drug–target interaction prediction. In the process of assessing performance, we document the average scores achieved by each prediction method. The specific definitions of the above mentioned metrics are as follows:

7 $$\begin{aligned}{} & {} \text {Precision} = \frac{TP}{TP + FP} \end{aligned}$$

8 $$\begin{aligned}{} & {} \text {Recall} = \frac{TP}{TP + FN} \end{aligned}$$

9 $$\begin{aligned}{} & {} \text {ACC} = \frac{TN + TP}{FP + TP + FN + TN} \end{aligned}$$

10 $$\begin{aligned}{} & {} \text {F1} = \frac{2 \cdot \text {Precision} \cdot \text {Recall}}{\text {Precision} + \text {Recall}} \end{aligned}$$

11 $$\begin{aligned}{} & {} \text {MCC} = \frac{TP \cdot TN - FP \cdot FN}{\sqrt{(TP + FP)(TP + FN)(TN + FP)(TN + FN)}} \end{aligned}$$

### Results

#### Comparison between DTIOG variants focusing on KGE

##### ENZ dataset using KGE

In Table 4, we find that DTIOG-DT consistently outperforms the competition across various similarity metrics. Notably, when assessed with the Cosine similarity metric, DTIOG-DT exhibits remarkable results with an AUPR that is 0.9% higher (0.976 vs. 0.967), an AUC that is 0.1% higher, an ACC that is 0.3% higher, an MCC that is 1.02% higher, and an F1 that is 0.92% higher than the next best classifier, DTIOG-ETC. This significant performance margin highlights the exceptional predictive capabilities of DTIOG-DT. Similarly, DTIOG-DT excels when evaluated using the Euclidean distance, Manhattan distance, Jaccard similarity, and Pearson correlation coefficient metrics, consistently achieving superior results compared to the other methods. DTIOG-ETC follows as the second-best classifier, delivering robust results across these similarity metrics, even though it trails slightly behind DTIOG-DT. In contrast, DTIOG-GradientBoosting, DTIOG-GaussianNB, and DTIOG-SGD consistently underperform, particularly when assessed using these similarity metrics, exhibiting lower in a diverse set of evaluation metrics values. For instance, DTIOG-GradientBoosting falls behind DTIOG-DT by 47.84% in AUPR, 16.06% in AUC, 9.25% in ACC, 57.20% in MCC, and 55.28% in F1 using Cosine similarity, indicating that these classifiers may not be suitable choices for the GPCR dataset. These findings underscore the importance of classifier selection in DTI prediction, with DTIOG-DT emerging as a top-performing option consistently.

**Table 4:** Performance comparison of DTIOG variants on the ENZ dataset using KGE

Approaches	AUPR	AUC	ACC	MCC	F1
*Cosine similarity*					
DTIOG-RF	0.963	0.994	0.991	0.958	0.962
DTIOG-DT	0.976	0.996	**0.994**	0.972	0.975
DTIOG-MLP	0.973	0.996	0.980	0.902	0.913
DTIOG-KNeighbors	0.973	0.996	0.968	0.869	0.879
DTIOG-BaggingClassifier	0.986	0.998	0.970	0.878	0.887
DTIOG-GradientBoosting	0.509	0.836	0.902	0.416	0.436
DTIOG-GaussianNB	0.586	0.840	0.848	0.452	0.513
DTIOG-SGD	0.661	0.922	0.921	0.574	0.608
DTIOG-ETC	0.967	0.995	0.991	0.962	0.966
*Euclidean distance*					
DTIOG-RF	0.964	0.995	0.991	0.959	0.963
DTIOG-DT	0.976	0.996	**0.994**	**0.973**	**0.976**
DTIOG-MLP	0.950	0.992	0.970	0.854	0.871
DTIOG-KNeighbors	0.974	0.996	0.970	0.876	0.885
DTIOG-BaggingClassifier	0.986	0.998	0.974	0.890	0.899
DTIOG-GradientBoosting	0.459	0.808	0.902	0.418	0.434
DTIOG-GaussianNB	0.583	0.833	0.831	0.423	0.486
DTIOG-SGD	0.656	0.922	0.919	0.561	0.595
DTIOG-ETC	0.967	0.995	0.992	0.963	0.966
*Manhattan distance*					
DTIOG-RF	0.965	0.995	0.991	0.960	0.964
DTIOG-DT	0.976	0.996	**0.994**	0.972	0.975
DTIOG-MLP	0.952	0.993	0.970	0.855	0.872
DTIOG-KNeighbors	0.981	0.997	0.974	0.893	0.902
DTIOG-BaggingClassifier	**0.989**	**0.998**	0.976	0.898	0.907
DTIOG-GradientBoosting	0.612	0.885	0.915	0.526	0.553
DTIOG-GaussianNB	0.584	0.838	0.838	0.440	0.502
DTIOG-SGD	0.662	0.923	0.917	0.554	0.585
DTIOG-ETC	0.969	0.995	0.992	0.964	0.968
*Jaccard similarity*					
DTIOG-RF	0.962	0.994	0.990	0.956	0.960
DTIOG-DT	0.975	0.996	0.993	0.971	0.974
DTIOG-MLP	0.956	0.993	0.971	0.855	0.870
DTIOG-KNeighbors	0.963	0.994	0.960	0.844	0.855
DTIOG-BaggingClassifier	0.979	0.997	0.963	0.852	0.862
DTIOG-GradientBoosting	0.606	0.879	0.914	0.512	0.539
DTIOG-GaussianNB	0.569	0.827	0.774	0.371	0.432
DTIOG-SGD	0.658	0.921	0.921	0.570	0.603
DTIOG-ETC	0.966	0.995	0.991	0.961	0.965
*Pearson correlation coefficient*					
DTIOG-RF	0.963	0.994	0.991	0.958	0.962
DTIOG-DT	0.975	0.996	**0.994**	0.971	0.974
DTIOG-MLP	0.973	0.996	0.980	0.906	0.917
DTIOG-KNeighbors	0.970	0.995	0.962	0.851	0.861
DTIOG-BaggingClassifier	0.985	**0.998**	0.965	0.860	0.869
DTIOG-GradientBoosting	0.650	0.900	0.920	0.556	0.583
DTIOG-GaussianNB	0.578	0.843	0.855	0.456	0.519
DTIOG-SGD	0.656	0.923	0.919	0.560	0.594
DTIOG-ETC	0.967	0.995	0.991	0.962	0.966

The best results are indicated in bold concerning each row

##### The GPCR dataset using KGE

In Table 5, we find that DTIOG-DT consistently outperforms the competition across various similarity metrics. Notably, when assessed with the Cosine similarity metric, DTIOG-DT exhibits remarkable results with an AUPR that is 0.61% higher, an AUC that is 0.3% higher, an ACC that is 0.4% higher, an MCC that is 0.83% higher, and an F1 that is 0.72% higher than the next best classifier, DTIOG-ETC. This significant performance margin highlights the exceptional predictive capabilities of DTIOG-DT.

**Table 5:** Performance comparison of DTIOG variants on the GPCR dataset using KGE

Approaches	AUPR	AUC	ACC	MCC	F1
*Cosine similarity*					
DTIOG-RF	0.951	0.980	0.971	0.931	0.949
DTIOG-DT	0.968	0.987	**0.981**	**0.955**	**0.967**
DTIOG-MLP	0.970	**0.992**	0.977	0.944	0.959
DTIOG-KNeighbors	0.949	0.979	0.899	0.792	0.843
DTIOG-BaggingClassifier	0.951	0.984	0.896	0.787	0.839
DTIOG-GradientBoosting	0.755	0.908	0.855	0.625	0.720
DTIOG-GaussianNB	0.655	0.784	0.716	0.405	0.586
DTIOG-SGD	0.617	0.822	0.775	0.438	0.574
DTIOG-ETC	0.962	0.984	0.977	0.947	0.960
*Euclidean distance*					
DTIOG-RF	0.956	0.982	0.974	0.938	0.954
DTIOG-DT	0.967	0.987	**0.981**	0.954	0.966
DTIOG-MLP	0.960	0.990	0.979	0.950	0.963
DTIOG-KNeighbors	0.946	0.977	0.901	0.797	0.846
DTIOG-BaggingClassifier	0.947	0.983	0.898	0.790	0.841
DTIOG-GradientBoosting	0.736	0.888	0.847	0.601	0.700
DTIOG-GaussianNB	0.653	0.778	0.709	0.388	0.575
DTIOG-SGD	0.589	0.811	0.765	0.417	0.555
DTIOG-ETC	0.964	0.985	0.979	0.949	0.962
*Manhattan distance*					
DTIOG-RF	0.953	0.980	0.972	0.933	0.951
DTIOG-DT	0.966	0.986	0.980	0.952	0.965
DTIOG-MLP	0.969	**0.992**	0.980	0.952	0.964
DTIOG-KNeighbors	0.963	0.985	0.908	0.808	0.855
DTIOG-BaggingClassifier	0.958	0.986	0.898	0.790	0.841
DTIOG-GradientBoosting	0.762	0.909	0.862	0.644	0.736
DTIOG-GaussianNB	0.646	0.781	0.719	0.418	0.594
DTIOG-SGD	0.599	0.817	0.777	0.435	0.570
DTIOG-ETC	0.966	0.986	0.980	0.952	0.965
*Jaccard similarity*					
DTIOG-RF	0.952	0.980	0.971	0.932	0.950
DTIOG-DT	0.966	0.986	0.980	0.953	0.965
DTIOG-MLP	0.936	0.982	0.960	0.906	0.931
DTIOG-KNeighbors	0.957	0.983	0.907	0.801	0.844
DTIOG-BaggingClassifier	**0.971**	0.990	0.911	0.813	0.859
DTIOG-GradientBoosting	0.779	0.920	0.868	0.660	0.748
DTIOG-GaussianNB	0.654	0.782	0.726	0.423	0.597
DTIOG-SGD	0.627	0.830	0.783	0.443	0.553
DTIOG-ETC	0.961	0.984	0.977	0.945	0.959
*Pearson correlation coefficient*					
DTIOG-RF	0.952	0.980	0.971	0.933	0.950
DTIOG-DT	0.965	0.986	0.980	0.951	0.964
DTIOG-MLP	0.965	0.991	0.980	0.951	0.964
DTIOG-KNeighbors	0.958	0.983	0.902	0.797	0.847
DTIOG-BaggingClassifier	0.957	0.986	0.892	0.780	0.834
DTIOG-GradientBoosting	0.754	0.907	0.856	0.628	0.723
DTIOG-GaussianNB	0.648	0.783	0.725	0.414	0.592
DTIOG-SGD	0.597	0.815	0.775	0.424	0.556
DTIOG-ETC	0.962	0.985	0.978	0.947	0.961

The best results are indicated in bold concerning each row

Similarly, DTIOG-DT consistently does better than the other methods when tested using the Euclidean distance, Manhattan distance, Jaccard similarity, and Pearson correlation coefficient metrics. DTIOG-ETC follows as the second-best classifier, delivering robust results across these similarity metrics, although it trails slightly behind DTIOG-DT.

On the other hand, DTIOG-GradientBoosting, DTIOG-GaussianNB, and DTIOG-SGD always do worse, especially when these similarity metrics are used to measure performance, showing lower values in a diverse set of evaluation metrics. For instance, DTIOG-GradientBoosting falls behind DTIOG-DT by 22% in AUPR, 8% in AUC, 12.84% in ACC, 34.55% in MCC, and 25.54% in F1 using Cosine similarity, indicating that these classifiers may not be suitable choices for the GPCR dataset.

##### The IC dataset using KGE

In Table 6, we find that DTIOG-DT consistently outperforms the competition across various similarity metrics. The Cosine similarity metric shows that DTIOG-DT does much better than the next best classifier, DTIOG-ETC. It has an AUPR that is 0.2% higher, an AUC that is 0.2% higher, an ACC that is 0.2% higher, an MCC that is 0.41% higher, and an F1 that is 0.2% higher. This significant performance margin highlights the exceptional predictive capabilities of DTIOG-DT. The Euclidean distance, Manhattan distance, Jaccard similarity, and Pearson correlation coefficient metrics also show that DTIOG-DT is very good. It consistently achieves superior results compared to the other methods, demonstrating higher values in a diverse set of evaluation metrics. DTIOG-ETC follows as the second-best classifier, delivering robust results across these similarity metrics, although it trails slightly behind DTIOG-DT. On the other hand, DTIOG-GradientBoosting, DTIOG-GaussianNB, and DTIOG-SGD always do worse, especially when these similarity metrics are used to measure them. For instance, DTIOG-GradientBoosting falls behind DTIOG-DT by 14.19% in AUPR, 6.18% in AUC, 11.91% in ACC, 28.98% in MCC, and 20.08% in F1 using Cosine similarity, indicating that these classifiers may not be suitable choices for the IC dataset.

**Table 6:** Performance comparison of DTIOG variants on the IC dataset using KGE

Approaches	AUPR	AUC	ACC	MCC	F1
*Cosine similarity*					
DTIOG-RF	0.960	0.980	0.973	0.941	0.958
DTIOG-DT	0.972	0.987	0.982	0.959	0.971
DTIOG-MLP	0.988	0.995	0.981	0.958	0.971
DTIOG-KNeighbors	0.987	0.994	0.955	0.904	0.932
DTIOG-BaggingClassifier	**0.989**	**0.996**	0.951	0.896	0.9272
DTIOG-GradientBoosting	0.834	0.926	0.865	0.681	0.776
DTIOG-GaussianNB	0.757	0.854	0.803	0.547	0.6914
DTIOG-SGD	0.767	0.895	0.828	0.597	0.715
DTIOG-ETC	0.970	0.985	0.980	0.955	0.969
*Euclidean distance*					
DTIOG-RF	0.962	0.981	0.974	0.944	0.960
DTIOG-DT	0.977	0.989	**0.985**	**0.967**	**0.977**
DTIOG-MLP	0.962	0.984	0.952	0.887	0.920
DTIOG-KNeighbors	0.986	0.993	0.955	0.904	0.932
DTIOG-BaggingClassifier	0.988	0.995	0.953	0.900	0.929
DTIOG-GradientBoosting	0.831	0.927	0.872	0.697	0.787
DTIOG-GaussianNB	0.757	0.855	0.798	0.543	0.690
DTIOG-SGD	0.753	0.885	0.813	0.552	0.669
DTIOG-ETC	0.973	0.987	0.982	0.961	0.973
*Manhattan distance*					
DTIOG-RF	0.962	0.982	0.975	0.944	0.961
DTIOG-DT	0.976	0.988	0.984	0.965	0.975
DTIOG-MLP	0.983	0.993	0.971	0.936	0.955
DTIOG-KNeighbors	0.985	0.993	0.957	0.908	0.935
DTIOG-BaggingClassifier	0.988	0.995	0.954	0.902	0.931
DTIOG-GradientBoosting	0.827	0.924	0.871	0.694	0.785
DTIOG-GaussianNB	0.771	0.863	0.801	0.555	0.699
DTIOG-SGD	0.752	0.883	0.815	0.560	0.683
DTIOG-ETC	0.973	0.987	0.982	0.960	0.972
*Jaccard similarity*					
DTIOG-RF	0.958	0.979	0.972	0.938	0.956
DTIOG-DT	0.973	0.987	0.982	0.961	0.973
DTIOG-MLP	0.981	0.993	0.980	0.955	0.969
DTIOG-KNeighbors	0.983	0.992	0.940	0.874	0.911
DTIOG-BaggingClassifier	0.981	0.992	0.931	0.858	0.900
DTIOG-GradientBoosting	0.834	0.928	0.867	0.685	0.778
DTIOG-GaussianNB	0.754	0.848	0.809	0.555	0.693
DTIOG-SGD	0.779	0.899	0.828	0.592	0.705
DTIOG-ETC	0.967	0.984	0.978	0.951	0.966
*Pearson correlation coefficient*					
DTIOG-RF	0.957	0.979	0.971	0.937	0.9560
DTIOG-DT	0.972	0.986	0.982	0.959	0.971
DTIOG-MLP	0.987	0.995	0.978	0.951	0.966
DTIOG-KNeighbors	0.984	0.992	0.945	0.883	0.918
DTIOG-BaggingClassifier	0.988	0.995	0.940	0.875	0.912
DTIOG-GradientBoosting	0.837	0.927	0.866	0.682	0.776
DTIOG-GaussianNB	0.757	0.849	0.799	0.542	0.689
DTIOG-SGD	0.770	0.894	0.824	0.601	0.724
DTIOG-ETC	0.968	0.985	0.979	0.953	0.967

The best results are indicated in bold concerning each row

### Comparison between DTIOG variants focused on KGE and ProtBERT

#### The ENZ dataset using ProtBERT

Table 7 depicts the performance of DTIOG variants using ProtBERT and the ENZ dataset. DTIOG-DT consistently performs better on a number of similarity metrics. Most notably, when appraised using the Cosine similarity metric, DTIOG-DT demonstrates exceptional results, with an AUPR that surpasses the closest competitor by 0.72%, an AUC that exceeds by 0.1%, an ACC that outperforms by 0.2%, an MCC that excels by 0.83%, and an F1 score that excels by 0.72%. This substantial performance differential underscores the outstanding predictive capabilities of DTIOG-DT.

**Table 7:** Performance comparison of DTIOG variants on the ENZ dataset using ProtBERT

Approaches	AUPR	AUC	ACC	MCC	F1
*Cosine similarity*					
DTIOG-RF	0.950	0.992	0.987	0.942	0.948
DTIOG-DT	0.963	0.994	0.990	0.957	0.961
DTIOG-MLP	0.701	0.928	0.924	0.590	0.621
DTIOG-KNeighbors	0.968	0.995	0.968	0.870	0.879
DTIOG-BaggingClassifier	**0.980**	**0.997**	0.978	0.906	0.914
DTIOG-GradientBoosting	0.455	0.808	0.898	0.384	0.402
DTIOG-GaussianNB	0.496	0.532	0.237	0.047	0.214
DTIOG-SGD	0.566	0.881	0.902	0.411	0.425
DTIOG-ETC	0.956	0.993	0.988	0.949	0.954
*Euclidean distance*					
DTIOG-RF	0.958	0.994	0.989	0.952	0.956
DTIOG-DT	0.971	0.996	**0.993**	0.967	0.970
DTIOG-MLP	0.944	0.991	0.968	0.844	0.861
DTIOG-KNeighbors	0.972	0.996	0.971	0.882	0.891
DTIOG-BaggingClassifier	0.978	0.992	0.971	0.898	0.906
DTIOG-GradientBoosting	0.644	0.908	0.918	0.548	0.578
DTIOG-GaussianNB	0.425	0.711	0.494	0.158	0.264
DTIOG-SGD	0.652	0.920	0.916	0.533	0.562
DTIOG-ETC	0.963	0.994	0.990	0.957	0.962
*Manhattan distance*					
DTIOG-RF	0.959	0.994	0.989	0.953	0.958
DTIOG-DT	0.973	0.996	**0.993**	**0.969**	**0.972**
DTIOG-MLP	0.964	0.995	0.975	0.882	0.893
DTIOG-KNeighbors	0.964	0.995	0.966	0.864	0.873
DTIOG-BaggingClassifier	0.976	**0.997**	0.973	0.889	0.898
DTIOG-GradientBoosting	0.380	0.698	0.895	0.311	0.278
DTIOG-GaussianNB	0.517	0.796	0.831	0.419	0.483
DTIOG-SGD	0.654	0.923	0.919	0.564	0.599
DTIOG-ETC	0.961	0.994	0.990	0.956	0.960
*Jaccard similarity*					
DTIOG-RF	0.943	0.991	0.985	0.933	0.939
DTIOG-DT	0.962	0.994	0.990	0.956	0.961
DTIOG-MLP	0.701	0.903	0.925	0.574	0.586
DTIOG-KNeighbors	0.969	0.995	0.970	0.877	0.886
DTIOG-BaggingClassifier	**0.980**	**0.997**	0.977	0.902	0.910
DTIOG-GradientBoosting	0.585	0.869	0.910	0.473	0.489
DTIOG-GaussianNB	0.490	0.539	0.254	0.045	0.213
DTIOG-SGD	0.579	0.872	0.906	0.434	0.440
DTIOG-ETC	0.954	0.993	0.988	0.946	0.951
*Pearson correlation coefficient*					
DTIOG-RF	0.950	0.992	0.987	0.942	0.947
DTIOG-DT	0.962	0.994	0.990	0.956	0.961
DTIOG-MLP	0.709	0.921	0.925	0.587	0.612
DTIOG-KNeighbors	0.967	0.995	0.968	0.869	0.879
DTIOG-BaggingClassifier	**0.980**	**0.997**	0.978	0.904	0.912
DTIOG-GradientBoosting	0.363	0.708	0.893	0.319	0.320
DTIOG-GaussianNB	0.493	0.528	0.235	0.038	0.211
DTIOG-SGD	0.567	0.880	0.901	0.422	0.446
DTIOG-ETC	0.954	0.993	0.988	0.947	0.952

The best results are indicated in bold concerning each row

Using the Euclidean distance, Manhattan distance, Jaccard similarity, and Pearson correlation coefficient metrics, DTIOG-DT always does better than other methods. DTIOG-ETC emerges as the second-best classifier, offering robust performance across these similarity metrics, albeit slightly trailing DTIOG-DT.

DTIOG-GradientBoosting, DTIOG-GaussianNB, and DTIOG-SGD, on the other hand, always do worse, especially when tested with these similarity metrics, giving lower values in a diverse set of evaluation metrics. For example, when assessed using the Cosine similarity, DTIOG-GradientBoosting lags behind DTIOGDT by 52.75% in AUPR, 18.71% in AUC, 9.29% in ACC, 59.87% in MCC, and 58.16% in F1. These results suggest that these classifiers may not be suitable choices for the ENZ dataset. The results make it clear how important it is to choose the right classifier for DTI prediction, with DTIOG-DT consistently coming out on top.

#### IC dataset using ProtBERT

As Table 8 depicts, DTIOG-DT consistently emerges as the top-performing classifier, showcasing its exceptional predictive capabilities. For instance, with the Cosine similarity metric, DTIOG-DT outperforms competitors, such as DTIOGETC, by 0.41% in AUPR, 0.2% in AUC, 0.2% in ACC, 0.63% in MCC, and 0.31% in F1. This trend holds true across other similarity metrics, underscoring DTIOG-DT’s robustness in a diverse set of evaluation metrics. As an alternative, DTIOG-ETC often comes in second place among classifiers, especially when it comes to Cosine similarity, Jaccard similarity, and Euclidean distance. The percentage differences between these tests and DTIOG-DT are significant, but they are also within a reasonable range. They are usually between 0.4% and 0.6% for AUPR, between 0.2% and 0.3% for AUC, between 0.3% and 0.4% for ACC, between 0.6% and 1.04% for MCC, and between 0.5% and 0.7% for F1. On the other side, it is worth mentioning that DTIOG-GradientBoosting, DTIOG-GaussianNB, and DTIOG-SGD are not very good at predicting across a number of similarity metrics. Compared to DTIOG-DT, they also have large negative percentage differences. For example, when using the Cosine similarity metric, DTIOG-GradientBoosting underperforms DTIOGDT by 19.58% in AUPR, 10.78% in AUC, 15.67% in ACC, 39.97% in MCC, and 28.76% in F1. This indicates that these classifiers may not be suitable choices for this particular task across the diverse set of evaluation metrics.

**Table 8:** Performance comparison of DTIOG variants on the IC dataset using ProtBERT

Approaches	AUPR	AUC	ACC	MCC	F1
*Cosine similarity*					
DTIOG-RF	0.951	0.976	0.966	0.927	0.949
DTIOG-DT	0.965	0.983	0.976	0.948	0.963
DTIOG-MLP	0.926	0.971	0.917	0.809	0.869
DTIOG-KNeighbors	0.974	0.987	0.932	0.860	0.901
DTIOG-BaggingClassifier	0.981	0.993	0.941	0.878	0.913
DTIOG-GradientBoosting	0.776	0.877	0.823	0.569	0.686
DTIOG-GaussianNB	0.659	0.779	0.785	0.458	0.523
DTIOG-SGD	0.687	0.816	0.775	0.451	0.600
DTIOG-ETC	0.961	0.981	0.974	0.942	0.960
*Euclidean distance*					
DTIOG-RF	0.956	0.978	0.970	0.934	0.954
DTIOG-DT	0.972	0.986	0.981	0.959	0.971
DTIOG-MLP	**0.997**	**0.999**	**0.995**	0.988	**0.992**
DTIOG-KNeighbors	0.971	0.986	0.971	0.936	0.955
DTIOG-BaggingClassifier	0.654	0.500	0.691	0.000	0.000
DTIOG-GradientBoosting	0.889	0.961	0.913	0.797	0.860
DTIOG-GaussianNB	0.713	0.817	0.784	0.492	0.647
DTIOG-SGD	0.745	0.877	0.816	0.566	0.696
DTIOG-ETC	0.966	0.983	0.977	0.949	0.964
*Manhattan distance*					
DTIOG-RF	0.957	0.979	0.971	0.936	0.955
DTIOG-DT	0.973	0.987	0.982	0.961	0.972
DTIOG-MLP	**0.997**	**0.999**	**0.995**	**0.989**	**0.992**
DTIOG-KNeighbors	0.977	0.989	0.960	0.914	0.940
DTIOG-BaggingClassifier	0.981	0.993	0.965	0.924	0.947
DTIOG-GradientBoosting	0.809	0.916	0.859	0.664	0.762
DTIOG-GaussianNB	0.694	0.800	0.780	0.471	0.622
DTIOG-SGD	0.749	0.875	0.813	0.561	0.692
DTIOG-ETC	0.970	0.985	0.980	0.956	0.969
*Jaccard similarity*					
DTIOG-RF	0.954	0.977	0.969	0.932	0.952
DTIOG-DT	0.969	0.985	0.979	0.954	0.968
DTIOG-MLP	0.988	0.995	0.976	0.945	0.962
DTIO-KNeighbors	0.979	0.990	0.948	0.889	0.922
DTIOGBaggingClassifier	0.991	0.996	0.960	0.913	0.939
DTIO-GradientBoosting	0.802	0.895	0.845	0.624	0.727
DTIO-GaussianNB	0.663	0.783	0.776	0.436	0.477
DTIO-SGD	0.722	0.846	0.800	0.518	0.648
DTIOG-ETC	0.965	0.983	0.976	0.948	0.963
*Pearson correlation coefficient*					
DTIOG-RF	0.955	0.978	0.970	0.933	0.953
DTIOG-DT	0.968	0.985	0.979	0.953	0.967
DTIOG-MLP	0.992	0.997	0.991	0.980	0.986
DTIOG-KNeighbors	0.982	0.991	0.955	0.904	0.932
DTIOG-BaggingClassifier	0.991	0.996	0.966	0.926	0.948
DTIOG-GradientBoosting	0.806	0.900	0.850	0.639	0.741
DTIOG-GaussianNB	0.672	0.791	0.785	0.458	0.523
DTIOG-SGD	0.738	0.858	0.810	0.541	0.669
DTIOG-ETC	0.968	0.984	0.979	0.953	0.967

The best results are indicated in bold concerning each row

#### The GPCR dataset using ProtBERT

In Table 9, when evaluating the performance of various classifiers on the GPCR dataset using the various similarity metrics, DTIOG-ETC emerges as the top performer, surpassing its competitors by significant percentages. DTIOG-DT achieves an AUPR that is 0.3% lower, an AUC that is 0.2% lower, an ACC that is 0.3% lower, an MCC that is 0.53% lower, and an F1 that is 0.42% lower than the first best classifier, DTIOG-ETC. This substantial performance margin underscores the exceptional predictive capabilities of DTIOG-ETC.

**Table 9:** Performance comparison of DTIOG variants on the GPCR dataset using ProtBERT

Approaches	AUPR	AUC	ACC	MCC	F1
*Cosine similarity*					
DTIOG-RF	0.930	0.969	0.956	0.899	0.925
DTIOG-DT	0.948	0.978	0.968	0.926	0.945
DTIOG-MLP	0.836	0.936	0.874	0.673	0.755
DTIOG-KNeighbors	0.946	0.977	0.939	0.865	0.899
DTIOG-BaggingClassifier	0.635	0.500	0.683	0.000	0.042
DTIOG-GradientBoosting	0.792	0.923	0.872	0.669	0.755
DTIOG-GaussianNB	0.431	0.628	0.439	0.108	0.437
DTIOG-SGD	0.454	0.691	0.720	0.274	0.443
DTIOG-ETC	0.951	0.980	0.971	0.931	0.949
*Euclidean distance*					
DTIOG-RF	0.950	0.979	0.970	0.930	0.948
DTIOG-DT	0.963	0.985	0.978	0.948	0.961
DTIOG-MLP	**0.995**	**0.998**	**0.993**	**0.983**	**0.987**
DTIOG-KNeighbors	0.961	0.984	0.959	0.905	0.930
DTIOG-BaggingClassifier	0.635	0.500	0.729	0.000	0.000
DTIOG-GradientBoosting	0.894	0.973	0.936	0.842	0.885
DTIOG-GaussianNB	0.597	0.772	0.752	0.436	0.604
DTIOG-SGD	0.594	0.805	0.784	0.437	0.572
DTIOG-ETC	0.957	0.982	0.974	0.940	0.955
*Manhattan distance*					
DTIOG-RF	0.950	0.979	0.970	0.930	0.948
DTIOG-DT	0.959	0.983	0.976	0.943	0.958
DTIOG-MLP	0.994	**0.998**	**0.993**	**0.983**	**0.987**
DTIOG-KNeighbors	0.959	0.983	0.953	0.894	0.921
DTIOG-BaggingClassifier	0.635	0.500	0.683	0.000	0.042
DTIOG-GradientBoosting	0.872	0.966	0.931	0.828	0.875
DTIOG-GaussianNB	0.586	0.775	0.755	0.436	0.602
DTIOG-SGD	0.591	0.803	0.781	0.426	0.568
DTIOG-ETC	0.960	0.983	0.976	0.944	0.958
*Jaccard similarity*					
DTIOG-RF	0.938	0.974	0.962	0.912	0.934
DTIOG-DT	0.958	0.983	0.975	0.941	0.956
DTIOG-MLP	0.984	0.996	0.989	0.974	0.981
DTIO-KNeighbors	0.968	0.987	0.965	0.918	0.939
DTIOGBaggingClassifier	0.635	0.500	0.729	0.000	0.000
DTIO-GradientBoosting	0.880	0.969	0.923	0.808	0.861
DTIO-GaussianNB	0.473	0.660	0.471	0.138	0.448
DTIO-SGD	0.560	0.780	0.775	0.400	0.536
DTIOG-ETC	0.954	0.981	0.972	0.934	0.951
*Pearson correlation coefficient*					
DTIOG-RF	0.940	0.975	0.963	0.915	0.937
DTIOG-DT	0.956	0.982	0.974	0.938	0.954
DTIOG-MLP	0.987	0.996	0.990	0.975	0.981
DTIOG-KNeighbors	0.972	0.989	0.961	0.910	0.933
DTIOG-BaggingClassifier	0.635	0.500	0.729	0.000	0.000
DTIOG-GradientBoosting	0.898	0.975	0.934	0.838	0.882
DTIOG-GaussianNB	0.497	0.676	0.527	0.189	0.468
DTIOG-SGD	0.587	0.797	0.780	0.419	0.554
DTIOG-ETC	0.958	0.983	0.975	0.940	0.956

The best results are indicated in bold concerning each row

The best classifier, DTIOG-ETC, comes in front with strong results: its AUPR is 0.3% higher, its AUC is 0.2% higher, its ACC is 0.3% higher, its MCC is 0.53% higher, and its F1 is 0.42% higher than DTIOGDT. Despite slightly higher values, DTIOG-ETC still demonstrates strong predictive performance, making it a reliable primary choice among the classifiers assessed in this dataset.

However, DTIOG-GradientBoosting, DTIOG-GaussianNB, and DTIOG-SGD consistently underperform, particularly when assessed with the various similarity metrics, exhibiting lower values in the diverse set of evaluation metrics. For instance, DTIOG-GradientBoosting falls behind DTIOG-DT by 16.45% in AUPR and 5.61% in AUC using Cosine similarity. These classifiers may not be suitable choices for the GPCR dataset, and these findings underscore the significance of classifier selection in predictive performance assessment.

### Comparison of DTIOG versus other DTI competitor prediction methods

To see how well the DTIOG stacks up against alternative approaches in Table 10, we chose the DTIOG-DT as it consistently shows itself to be the best classifier among the different versions of DTIOG. We have applied DTIOG-DT in three different modes, depending on the contextual and local strategies. DTIOG-DT-KGE utilizes KGE for drugs and targets; DTIOG-DT-PRTB employs KGE for drugs and ProtBERT for targets in the contextual strategy. DTIOG-DT-FP figures out molecular fingerprints for drugs as part of the local strategy, and for proteins, sequence characteristics are used. Additionally, for each submode, various similarity metrics were taken into consideration. In this order, these groups used Cosine similarity, Euclidian distance, Manhattan distance, Jaccard similarity, and Pearson correlation, named as DTIOG-DT-KGE-COS, DTIOG-DT-KGE-ED, DTIOG-DT-KGE-MD, DTIOG-DT-KGE-JCC, and DTIOG-DT-KGE-PCC, respectively.

**Table 10:** Comparison of DTIOG variants versus other DTI competitor prediction methods

Classifiers	AUPR	AUC	ACC	MCC	F1
*GPCR*					
DTIOG-DT-PRTB-COS	0.948	0.978	0.968	0.926	0.945
DTIOG-DT-PRTB-ED	0.963	0.985	0.978	0.948	0.961
DTIOG-DT-PRTB-MD	0.959	0.983	0.976	0.943	0.958
DTIOG-DT-PRTB-JCC	0.958	0.983	0.975	0.941	0.956
DTIOG-DT-PRTB-PCC	0.956	0.982	0.974	0.938	0.954
DTIOG-DT-KGE-COS	**0.968**	**0.987**	**0.981**	**0.955**	**0.967**
DTIOG-DT-KGE-ED	0.967	**0.987**	**0.981**	0.954	0.966
DTIOG-DT-KGE-MD	0.966	0.986	0.980	0.952	0.965
DTIOG-DT-KGE-JCC	0.966	0.986	0.980	0.953	0.965
DTIOG-DT-KGE-PCC	0.965	0.986	0.980	0.951	0.964
DTIOG-DT-FP-COS	0.953	0.980	0.971	0.933	0.950
DTIOG-DT-FP-ED	0.951	0.980	0.971	0.931	0.949
DTIOG-DT-FP-MD	0.953	0.981	0.972	0.934	0.951
DTIOG-DT-FP-JCC	0.954	0.981	0.972	0.933	0.951
DTIOG-DT-FP-PCC	0.957	0.982	0.974	0.940	0.955
iGRLDTI	0.954	0.979	0.944	0.850	0.885
DTI-HeNE	0.948	0.945	0.972	0.923	0.939
ALADIN	0.516	0.795	0.970	0.381	0.298
BLM-NII	0.476	0.834	0.970	0.297	0.189
*IC*					
DTIOG-DT-PRTB-COS	0.965	0.983	0.976	0.948	0.963
DTIOG-DT-PRTB-ED	0.972	0.986	0.981	0.959	0.971
DTIOG-DT-PRTB-MD	0.973	0.987	0.982	0.961	0.972
DTIOG-DT-PRTB-JCC	0.969	0.985	0.979	0.954	0.968
DTIOG-DT-PRTB-PCC	0.968	0.985	0.979	0.953	0.967
DTIOG-DT-KGE-COS	0.972	0.987	0.982	0.959	0.971
DTIOG-DT-KGE-ED	0.977	**0.989**	0.985	**0.967**	**0.977**
DTIOG-DT-KGE-MD	0.976	0.988	0.984	0.965	0.975
DTIOG-DT-KGE-JCC	0.973	0.987	0.982	0.961	0.973
DTIOG-DT-KGE-PCC	0.972	0.986	0.982	0.959	0.971
DTIOG-DT-FP-COS	0.959	0.980	0.973	0.940	0.958
DTIOG-DT-FP-ED	0.962	0.981	0.974	0.944	0.960
DTIOG-DT-FP-MD	0.961	0.981	0.973	0.942	0.959
DTIOG-DT-FP-JCC	0.960	0.981	0.973	0.941	0.959
DTIOG-DT-FP-PCC	0.962	0.982	0.975	0.944	0.961
iGRLDTI	0.973	0.980	0.931	0.861	0.921
DTI-HeNE	**0.981**	0.978	**0.986**	0.964	0.973
ALADIN	0.803	0.913	0.965	0.757	0.751
BLM-NII	0.786	0.930	0.965	0.763	0.762
*ENZ*					
DTIOG-DT-PRTB-COS	0.963	0.994	0.990	0.957	0.961
DTIOG-DT-PRTB-ED	0.971	**0.996**	0.993	0.967	0.970
DTIOG-DT-PRTB-MD	0.973	**0.996**	0.993	0.969	0.972
DTIOG-DT-PRTB-JCC	0.962	0.994	0.990	0.956	0.961
DTIOG-DT-PRTB-PCC	0.962	0.994	0.990	0.956	0.961
DTIOG-DT-KGE-COS	**0.976**	**0.996**	**0.994**	0.972	0.975
DTIOG-DT-KGE-ED	**0.976**	**0.996**	**0.994**	**0.973**	**0.976**
DTIOG-DT-KGE-MD	**0.976**	**0.996**	**0.994**	0.972	0.975
DTIOG-DT-KGE-JCC	0.975	**0.996**	0.993	0.971	0.974
DTIOG-DT-KGE-PCC	0.975	**0.996**	**0.994**	0.971	0.974
DTIOG-DT-FP-COS	0.954	0.993	0.988	0.947	0.952
DTIOG-DT-FP-ED	0.951	0.992	0.987	0.943	0.948
DTIOG-DT-FP-MD	0.956	0.993	0.989	0.949	0.954
DTIOG-DT-FP-JCC	0.955	0.993	0.988	0.948	0.953
DTIOG-DT-FP-PCC	0.955	0.993	0.988	0.948	0.953
iGRLDTI	0.949	0.940	0.885	0.760	0.906
DTI-HeNE	0.963	0.962	0.992	0.954	0.957
ALADIN	0.757	0.896	0.990	0.665	0.620
BLM-NII	0.769	0.925	0.990	0.702	0.666

The best results are indicated in bold concerning each row

Table 10 depicts the performance of various DTIs prediction methods using diverse evaluation metrics across three DTI classes: GPCR, IC, and ENZ. The evaluated prediction methods include different variants of DTIOG, iGRLDTI, DTI-HeNE, ALADIN, and BLM-NII. In all three DTI classes, DTIOG variants consistently do better than iGRLDTI, DTI-HeNE, ALADIN, and BLM-NII in a number of evaluation metrics. Notably, DTIOG variants that use KGE have very good predictive power, as shown by their high AUPR scores. In particular, DTIOG-DT-KGE-COS, DTIOG-DT-KGE-ED, DTIOG-DT-KGE-MD, and DTIOG-DT-KGE-JCC excel at achieving the highest AUPR scores across various classes. To sum up, DTIOG is the best way to predict DTI because it can be used in a number of different ways, including using latent representations, feature representations of drugs and proteins, and different similarity metrics. Variants that use KGE work especially well, showing how important it is to represent DTIs in a latent space. Despite being a respectable method, iGRLDTI fails to outperform DTIOG across most metrics, underscoring the power of latent representation-based approaches. If we compare ALADIN and BLM-NII to DTIOG and other top methods, they do not do nearly as well. This shows how important it is to combine knowledge and latent representations for accurate DTI prediction.

DTIOG does much better than its competitors because it uses KGE to show complicated connections in KGs, which makes it better at modeling complicated drug-protein interactions. Adding to that, the novel approach, can extract features for both drugs and proteins. This gives a more complete picture of DTIs by looking at structural and sequential features.

#### Case study

The aim of our case study is to evaluate the practical efficacy of the DTIOG-BaggingClassifier variant using the KGE in identifying unknown DTIs. This classifier demonstrated strong performance during the training process, as indicated in Tables 4, 5, 6, and 7, providing well-validated predictions in terms of AUPR or AUC metrics.

Concerning prediction scores, the top 10 pairs in the testing dataset were selected for further validation. Each pair of drug and target was then verified against the latest versions of the DrugBank [44], CTD [61], KEGG [64] and ChEMBL [65] databases. These verified drug–target pairs did not exist in KG input during the embedding process or the training of the DTIOG variants. Instead, they were later added to the latest version of databases. Additionally, the DTIs for the different datasets (i.e., ENZ, GPCR, and IC datasets) were collected before 2008 [58], allowing verification using newly updated DTIs in the aforementioned databases.

Tables 11, 12, and 13 present the top 10 pairs of drugs and targets from the ENZ, GPCR, and IC datasets, respectively, with the highest prediction scores. Following a meticulous literature review of the predicted DTIs for the ENZ dataset, five of them were confirmed by the latest versions of the DrugBank, KEGG, CTD, or ChEMBL databases. Interestingly, in the GPCR dataset, the DTIOG-BaggingClassifier performed well by predicting 10 confirmed DTIs validated by DrugBank or KEGG databases. On the other hand, for the IC dataset, our approach successfully predicted six confirmed DTIs validated by DrugBank or KEGG databases.

**Table 11:** Top 10 predicted novel interactions in the ENZ dataset performed by DTIOG-BaggingClassifier, with supporting evidence from external databases

Rank	Drugbank ID	Drug name	Uniprot ID	Target name	Evidence
1	DB00586	Diclofenac	P05164	Myeloperoxidase	CTD
2	DB00312	Pentobarbital	P12931	Proto-oncogene tyrosine-protein kinase Src	KEGG
3	DB00201	Caffeine	P20853	Cytochrome P450 2A7	None
4	DB00312	Pentobarbital	P06276	Cholinesterase	None
5	DB00201	Caffeine	P14060	3 beta-hydroxysteroid dehydrogenase/Delta 5–>4-isomerase type 1	CTD
6	DB00201	Caffeine	Q13946	High affinity cAMP-specific 3’,5’-cyclic phosphodiesterase 7A	DrugBank
7	DB00564	Carbamazepine	Q92813	Type II iodothyronine deiodinase	None
8	DB01907	NADH	O94788	Retinal dehydrogenase 2	None
9	DB00432	Trifluridine	Q92813	Type II iodothyronine deiodinase	None
10	DB01115	Nifedipine	P11712	Cytochrome P450 2C9	DrugBank, ChEMBL

**Table 12:** Top 10 predicted novel interactions in the GPCR dataset performed by DTIOG-BaggingClassifier, with supporting evidence from external databases

Rank	Drugbank ID	Drug name	Uniprot ID	Target name	**Evidence**
1	DB00810	Biperiden	P20309	Muscarinic acetylcholine receptor M3	KEGG
2	DB00726	Trimipramine	P08912	Muscarinic acetylcholine receptor M5	DrugBank
3	DB00850	Perphenazine	P21918	D(1B) dopamine receptor	KEGG
4	DB00568	Cinnarizine	P08173	Muscarinic acetylcholine receptor M4	DrugBank, KEGG
5	DB00726	Trimipramine	P28223	5-hydroxytryptamine receptor 2A	DrugBank, KEGG
6	DB00568	Cinnarizine	P20309	Muscarinic acetylcholine receptor M3	DrugBank, KEGG
7	DB00462	Methscopolamine bromide	P20309	Muscarinic acetylcholine receptor M3	DrugBank, KEGG
8	DB01239	Chlorprothixene	P20309	Muscarinic acetylcholine receptor M3	DrugBank, KEGG
9	DB00933	Mesoridazine	P20309	Muscarinic acetylcholine receptor M3	KEGG
10	DB00454	Meperidine	Q9H3N8	Histamine H4 receptor	KEGG

**Table 13:** Top 10 predicted novel interactions in the IC dataset performed by DTIOG-BaggingClassifier, with supporting evidence from external databases

Rank	Drugbank ID	Drug name	Uniprot ID	Target name	Evidence
1	DB00653	Magnesium sulfate	Q01668	Voltage-dependent L-type calcium channel subunit alpha-1D	DrugBank
2	DB00349	Clobazam	P47870	Gamma-aminobutyric acid receptor subunit beta-2	DrugBank, KEGG
3	DB00818	Propofol	O75311	Glycine receptor subunit alpha-3	KEGG
4	DB01122	Ambenonium	P46098	5-hydroxytryptamine receptor 3A	None
5	DB01239	Chlorprothixene	Q92952	Small conductance calcium-activated potassium channel protein 1	None
6	DB00312	Pentobarbital	Q15878	Voltage-dependent R-type calcium channel subunit alpha-1E	None
7	DB00829	Diazepam	O75311	Glycine receptor subunit alpha-3	KEGG
8	DB00653	Magnesium sulfate	Q00975	Voltage-dependent N-type calcium channel subunit alpha-1B	None
9	DB00740	Riluzole	Q13002	Glutamate receptor ionotropic, kainate 2	KEGG
10	DB00949	Felbamate	O75311	Glycine receptor subunit alpha-3	KEGG

Regarding the prediction results, we find that the DTIOG-BaggingClassifier could predict validated DTIs, most of which are related to COVID-19, such as the predicted interaction between the drug Pentobarbital (i.e., DB00312) and the target Proto-oncogene tyrosine-protein kinase Src (i.e., P12931) (cf. Table 11). Barbiturate drugs like pentobarbital are mostly used as sedatives, hypnotics, or anesthetics. They work on the central nervous system by increasing the calming effects of the neurotransmitter gamma-aminobutyric acid (GABA) [66]. The proto-oncogene tyrosine-protein kinase Src target is a member of the tyrosine-protein kinase class, playing a pivotal role in cell signaling and regulation. While these kinases are vital for normal cellular processes, mutations or overexpression can render them oncogenic, thereby contributing to cancer development.

Furthermore, our approach has identified a drug–target interaction involving Caffeine (DB00201) and 3 beta-hydroxysteroid dehydrogenase/Delta 5–>4-isomerase type 1 (P14060) (cf. Table 11). Many studies, including [67–69], have shown that caffeine can effectively reduce inflammation and change the way the immune system works. In the airway smooth muscle, it exerts bronchodilator effects primarily through its role as a phosphodiesterase inhibitor and adenosine receptor antagonist. On the other hand, the enzyme P14060, encoded by the gene HSD3B1, plays a vital role in the biosynthesis of steroid hormones. Specifically, it is instrumental in converting pregnenolone to progesterone and actively contributes to the production of diverse steroid hormones. Its significance extends to the synthesis of various steroid hormones, particularly those crucial for the reproductive system and stress response.

Additionally, the DTIOG variant has the capability to predict an interaction between Nifedipine (DB01115) and the target Cytochrome P450 2C9 (P11712) (cf. Table 11). Nifedipine is a calcium channel blocker that is mostly used to treat angina and high blood pressure. It works by stopping calcium ions from entering the heart and smooth muscle cells, which opens up blood vessels and lowers the heart’s workload. Nifedipine could potentially serve as a therapeutic molecule for managing the pathophysiological conditions of the lungs in severe COVID-19 patients [70, 71]. Cytochrome P450 2C9 (CYP2C9), a member of the cytochrome P450 enzyme family, plays a crucial role in drug metabolism. Predominantly located in the liver, CYP2C9 is responsible for metabolizing a wide range of drugs, including Nifedipine. The interaction between Nifedipine and CYP2C9 involves the enzyme’s function in breaking down the drug, thereby influencing its pharmacokinetics.

In particular, we notice that DTIOG successfully identifies the interaction between the drug Cinnarizine (DB00568) and the target protein Muscarinic acetylcholine receptor M4 (P08173) (cf. Table 12). Cinnarizine has demonstrated positive outcomes in patients with COVID-19-associated CLLs, likely owing to its antihistaminic and calcium channel-blocking properties [72]. On the other hand, P08173 is a G-protein-coupled receptor activated by the neurotransmitter acetylcholine, predominantly located in the central nervous system. Upon activation, it can modulate various physiological processes.

A new interaction has been found between the drug magnesium sulfate (DB00653) and the target protein voltage-dependent L-type calcium channel subunit alpha-1D (Q01668) (cf. Table 13). There are several ways that magnesium sulfate protects organs and tissues from damage. These include reducing inflammation, fighting free radicals, and keeping the immune system in check [73, 74]. Q01668 is a protein intricately involved in the regulation of calcium ion flow across cell membranes. In addition, an interaction has been observed between Propofol (DB00818) and the target Glycine receptor subunit alpha-3 (O75311) (cf. Table 13). Propofol is a potent intravenous anesthetic commonly used for the induction and maintenance of general anesthesia. Hypertriglyceridemia frequently occurs in COVID-19 patients receiving propofol, but it does not lead to acute pancreatitis. On the other hand, O75311 is a component of the glycine receptor, a ligand-gated ion channel widely distributed in the central nervous system. It plays a crucial role in mediating inhibitory neurotransmission, particularly in the spinal cord and brainstem.

Therefore, DTIOG proves to be a valuable tool for uncovering new drug–target interactions, particularly in cases where drugs are associated with COVID-19. This guess comes from adding to the KG a list of genes (e.g., CCL2, TNF, and IL6), drugs (e.g., Ruxolitinib, Choline, Chloroquine, and Baricitinib), and gene ontology terms (e.g., cell proliferation and response to oxidative stress) that are linked to COVID-19 from the CTD database [61]. Additionally, we compute all interactions between SARS-CoV-2 and Homo sapiens proteins. Moreover, we annotate each SARS-CoV-2 protein with its gene ontology terms.

### Discussion

The approach employed in our study utilizes the interconnected network of drug–target relationships within the KG to generate precise predictions pertaining to potential DTIs. The graph facilitates the analysis of direct interactions and the investigation of indirect associations. For example, pharmaceutical substances have the ability to selectively bind to proteins that are part of the same biological pathway or possess similar molecular structures. This characteristic enhances the probability of interaction between the drugs and their target proteins. Due to the extensive network of connections within the KG, our approach is capable of leveraging the comprehensive knowledge repository to accurately forecast potential interactions between a drug and a target.

Various types of information have been extracted from the knowledge graph, encompassing drug and target embedding vectors as well as two similarity matrices: drug–drug and target–target. Our methodology involves addressing these constituent elements separately, thereby facilitating a more focused examination of the distinct attributes and interconnections of each entity. By examining the embeddings of drugs and targets, as well as their respective similarity matrices, it becomes more feasible to identify underlying associations and potential interactions. Furthermore, our methodology employs various representations and similarity matrices to effectively capture the inherent properties and specific associations between drugs and targets. This facilitates the comprehension of the predictions. The utilization of this dual approach allows researchers to gain a comprehensive understanding of the various factors that influence DTIs.

Utilizing drug and target embeddings in conjunction with drug–drug and target–target similarity matrices constitutes a viable approach for predicting the interaction between a drug and its target. These methodologies offer a more intricate comprehension of drugs and their intended targets, facilitate the detection of hidden associations, and augment the comprehensibility of predictions. Consequently, they contribute to the progression of our understanding in the realm of pharmaceutical exploration and advancement. The primary strength of our methodology resides in its capacity to integrate pre-existing high-order proximity data regarding drugs and targets, resulting in representations of drug–target pairs. Additionally, our approach offers the flexibility to modify the length of these representations in order to fulfill specific task demands. The utilization of an integration-based algorithm as the core method for processing the heterogeneous DTIs network leads to these advantages.

Using much more information about similar things, the principle mentioned earlier can help predict unknown DTIs more purposefully and directly, reducing the likelihood of making mistakes. Nevertheless, this approach has the disadvantage of narrowing down the search space for novel DTIs. Suppose the similarity between the nodes of a particular drug–target pair and other nodes in the dataset is relatively low. In that case, the probability of predicting a potential interaction between them is reduced, even if such an association exists.

To overcome this limitation, we intend to investigate how to extend our method functionally and assign greater attention to certain drugs with lower similarity to other drugs that still warrant further analysis. This course of action is expected to broaden the range of potential DTIs that our method can uncover. Furthermore, our proposed methodology employs a sequential transductive learning framework for the prediction of DTIs. This approach enhances comprehensibility as each step within the workflow possesses a distinct and unambiguous interpretation. Nevertheless, the current transductive-style operation employed by our method results in increased computational expenses in comparison to inductive learning methods.

Inductive learning techniques exhibit no specific constraints regarding the dataset about fixed drugs and targets. On the other hand, transductive learning methods can enhance predictive accuracy by utilizing additional information from unknown samples in datasets with sparsely known interactions. However, any new nodes or samples added to the dataset necessitate the re-running of the model, which is a trade-off for the improved predictive accuracy provided by the transductive learning approach.

Our goal is to expand our approach to focus on drugs that have lower similarity to other drugs but still have potential for further investigation. In addition, our approach offers improved clarity by using a step-by-step transductive learning process. However, it does require more computational resources compared to inductive learning methods.

It is crucial to emphasize that integrating several similarity matrices from other approaches, such as the Node2vec approach, into our case is not recommended. The reason is that Node2vec relies solely on a single type of relationship, which is a connected relationship. However, our knowledge graph construction requires an embedding approach based on the triplets formed between various entity pairs. We have computed diverse types of similarities between drugs, primarily based on their chemical structure, including the SDF, MOL, or SMILES formats. Additionally, we can utilize different drug similarity measures based on side effects, such as Kuhn’s method, AERS-freq, and AERS-bit.

We have devised an approach to infer novel drugs from the KGE, employing diverse features and decomposition data with multiple classifiers. This approach can be extended to explore HP-PPI between SARS-CoV-2 and human proteins. To enrich our knowledge graph, we integrate information about the Mus-musculus species. Mouse models are widely used to assess COVID-19 disease risks and evaluate potential COVID-19 vaccines. Additionally, mouse models have proven valuable for drug development and studying various immune responses. Our strategy aims to establish an alignment between the PPI networks of Homo sapiens, Mus musculus, and the coronavirus species to identify potential viral interactions relevant to COVID-19. Moreover, we can utilize this alignment to investigate virus-host PPI networks between Homo sapiens, SARS-CoV-2, and SARS-CoV-1 proteins, thereby discovering more conserved edges or common viral interactions.

In the forthcoming network paradigm, our objective is to investigate both topological and biological hypotheses that arise from the interactions among biological entities. This exploration involves the application of representation learning techniques and clustering algorithms [39, 75]. Our future focus lies in leveraging the concept of multi-objective particle swarm optimization [75] to enhance the precision of DTI prediction models. More specifically, our approach involves the utilization of KGE techniques to generate drug embeddings and ProtBERT to obtain protein embeddings. In order to accomplish this objective, we suggest the incorporation of clustering algorithms such as FCAN-MOPSO [75] to facilitate the extraction of meaningful patterns in the data. FCAN-MOPSO is an enhanced graph clustering algorithm for complex networks using fuzzy logic and multi-objective particle swarm optimization. This approach can be applied to group similar drugs or proteins together. Once the clustering is performed, we can analyze the resulting clusters to gain insights into the biological relevance of the identified groups. For example, we can use enrichment analysis to find biological pathways or gene-ontology terms that are linked to proteins in each cluster. This step helps in understanding the functional context of the clustered proteins and their relationships to specific drugs. This approach enables a systematic exploration of DTIs in biological networks, providing valuable information for drug discovery and repurposing efforts. Meanwhile, the clustering analyses permit handling the cold-start problem, e.g., in the case of SARS-CoV-2 and its variants, when the approach needs to make predictions or recommendations for new or previously unseen drugs or targets for which there is limited or no existing data or associations available.

## Conclusion

The performance of DTIOG in predicting DTIs outperforms that of other existing approaches. The utilization of drug and target embeddings, along with similarity matrices, enhances the efficacy of the approach by facilitating interpretable predictions and fostering a thorough understanding of drug–target associations. The utilization of DTIOG holds substantial promise in enhancing endeavors related to drug discovery and development, and it may offer valuable insights in the exploration of HP-PPI for infectious diseases such as COVID-19. Our research not only highlights the effectiveness of our proposed approach but also emphasizes the crucial importance of graph-based methods and advanced contextual embeddings. This indicates a promising avenue for future research in the field of computational prediction of drug–target interactions.

## Data Availability

The source code of DTIOG and the datasets used to generate the results is accessible through https://doi.org/10.5281/zenodo.10209331.

## Abbreviations

DTIOG: Drug target interaction from knowledge graph
KGE: Knowledge graph embedding
PPIs: Protein–protein interactions
KGs: Knowledge graphs
HP-PPI: Host-pathogen protein–protein interactions
ML: Machine learning
DL: Deep learning
PU: Positive-unlabeled
DTIs: Drug–target interactions
SMILES: Simplified molecular input line entry specification
CMNMF: Constrained multi-view nonnegative matrix factorization
BERT: Bidirectional encoder representations from transformers
WkNN: Weighted k-nearest neighbors
HBIN: Heterogeneous biological information network
MLM: Masked language modeling
GCNs: Graph convolutional network
GBDT: Gradient boosting decision tree
RF: Random forest classifier
DT: Decision trees
MLP: Multi-layer perceptron
GaussianNB: Gaussian Naive Bayes
SGD: Stochastic gradient descent
ETC: Extra trees classifier
ENZ: Enzymes
IC: Ion channels
GPCR: G-protein-coupled receptors
AUPR: The area under the precision-recall curve
AUC: The area under the ROC curve
ACC: Accuracy
MCC: Matthew’s correlation coefficient
F1: F1-score
ProtBERT: Protein bidirectional encoder representations from transformers

## Declarations

### Availability and requirements

- Project name: DTIOG
- Project home page: https://doi.org/10.5281/zenodo.10209331
- Operating system(s): Linux
- Programming language: python
- License: BSD 3-Clause
- Any restrictions to use by non-academics: N/A
